# Cyclized NDGA modifies dynamic α-synuclein monomers preventing aggregation and toxicity

**DOI:** 10.1038/s41598-019-39480-z

**Published:** 2019-02-27

**Authors:** Malcolm J. Daniels, J. Brucker Nourse, Hanna Kim, Valerio Sainati, Marco Schiavina, Maria Grazia Murrali, Buyan Pan, John J. Ferrie, Conor M. Haney, Rani Moons, Neal S. Gould, Antonino Natalello, Rita Grandori, Frank Sobott, E. James Petersson, Elizabeth Rhoades, Roberta Pierattelli, Isabella Felli, Vladimir N. Uversky, Kim A. Caldwell, Guy A. Caldwell, Edward S. Krol, Harry Ischiropoulos

**Affiliations:** 1 Pharmacology Graduate Group, Raymond and Ruth Perelman School of Medicine, University of Pennsylvania, Philadelphia, PA 19104 USA; 2 Department of Biological Sciences, University of Alabama, Tuscaloosa, AL 35487 USA; 3 CERM and Department of Chemistry “Ugo Schiff”, University of Florence, Sesto Fiorentino, Florence 50019 Italy; 4 Department of Chemistry, University of Pennsylvania, Philadelphia, PA 19104 USA; 5 Department of Chemistry, University of Antwerp, Antwerp, Belgium; 6 Department of Pediatrics, Children’s Hospital of Philadelphia Research Institute, Philadelphia, PA 19104 USA; 7 Department of Biotechnology and Biosciences, University of Milan-Bicocca, Milan, Italy; 8 Biomolecular & Analytical Mass Spectrometry, Antwerp University, Antwerp, Belgium; 9 Astbury Centre for Structural Molecular Biology, University of Leeds, Leeds, United Kingdom; 10 School of Molecular and Cellular Biology, University of Leeds, Leeds, United Kingdom; 11 Department of Molecular Medicine and Byrd Alzheimer’s Research Institute, Morsani College of Medicine, University of South Florida, Tampa, FL 33612 USA; 12 Institute for Biological Instrumentation, Russian Academy of Sciences, Pushchino, Moscow Region 142292 Russian Federation; 13 College of Pharmacy & Nutrition, University of Saskatchewan, Saskatoon, Saskatchewan Canada; 14 Children’s Hospital of Philadelphia Research Institute and Systems Pharmacology and Translational Therapeutics, the Raymond and Ruth Perelman School of Medicine, University of Pennsylvania, Philadelphia, PA 19104 USA

## Abstract

Growing evidence implicates α-synuclein aggregation as a key driver of neurodegeneration in Parkinson’s disease (PD) and other neurodegenerative disorders. Herein, the molecular and structural mechanisms of inhibiting α-synuclein aggregation by novel analogs of nordihydroguaiaretic acid (NDGA), a phenolic dibenzenediol lignan, were explored using an array of biochemical and biophysical methodologies. NDGA analogs induced modest, progressive compaction of monomeric α-synuclein, preventing aggregation into amyloid-like fibrils. This conformational remodeling preserved the dynamic adoption of α-helical conformations, which are essential for physiological membrane interactions. Oxidation-dependent NDGA cyclization was required for the interaction with monomeric α-synuclein. NDGA analog-pretreated α-synuclein did not aggregate even without NDGA-analogs in the aggregation mixture. Strikingly, NDGA-pretreated α-synuclein suppressed aggregation of naïve untreated aggregation-competent monomeric α-synuclein. Further, cyclized NDGA reduced α-synuclein-driven neurodegeneration in *Caenorhabditis elegans*. The cyclized NDGA analogs may serve as a platform for the development of small molecules that stabilize aggregation-resistant α-synuclein monomers without interfering with functional conformations yielding potential therapies for PD and related disorders.

## Introduction

Parkinson’s disease (PD) is an age-related neurodegenerative disorder characterized by a progressive motor phenotype including tremors, rigidity, and bradykinesia. These symptoms are driven primarily by loss of dopamine-producing neurons in the *substantia nigra pars compacta*. Lewy bodies, intracellular proteinaceous inclusions, are the histopathological hallmark of PD. Immunohistochemical analysis of Lewy bodies revealed aggregated forms of α-synuclein, a 140 amino acid protein, as a major component^1,2^. Mutations, duplications, and triplications of the gene encoding α-synuclein cause dominantly-inherited familial forms of PD^3–6^. Furthermore, aggregation of wild type α-synuclein is involved in the pathogenesis of a diverse group of neurodegenerative diseases^1,7–14^.

The pathological and genetic evidence implicating α-synuclein in neurodegeneration has sparked numerous studies of the normal function of α-synuclein and its role in disease pathogenesis. α-Synuclein has been implicated in synaptic function^15^. It preferentially binds lipid membranes with high curvature^16^ and may modulate neurotransmitter release and synaptic function by affecting vesicular dynamics^17–21^.

Repeated studies across various animal models have shown that expression of mutant α-synuclein with altered aggregation kinetics causes neurodegeneration^22,23^. Likewise, induction of α-synuclein aggregation in wildtype animals by seeding with α-synuclein aggregates—isolated from PD Lewy bodies^24^ or generated *in vitro*^25–28^—induces progressive neurodegeneration. Indeed, this propagation of α-synuclein aggregation is not only observed in animal models, but in dopaminergic tissue grafted into PD patients^29^. α-Synuclein aggregation also occurs in animal models of dopamine neuron degeneration induced by oxidative chemical insult^30,31^.

The implication of α-synuclein in PD pathogenesis stimulated several screens for small molecules that alter its aggregation. Dopamine and related catecholamines were among the first molecules found to prevent α-synuclein fibril formation^32^. Oxidation of vicinal hydroxyls in dopamine, and related phenols, induces formation of soluble α-synuclein oligomers that do not incorporate into fibrils^33–35^. Recently, dopamine-induced α-synuclein oligomers were shown to contribute to neurodegeneration in mice^36^, but the precise mechanism of oligomer toxicity remains a subject of debate^37^. Dopamine oxidation may also contribute to neurodegeneration through modification of glucocerebrosidase and consequential lysosomal dysregulation^38^.

Ongoing research has identified many other inhibitors of α-synuclein aggregation. Many of these inhibitors stabilize multimers or oligomers through direct interaction with α-synuclein (e.g. various phenols, catechols, and flavonoids^35,39–45^, Anle138b^46,47^, rifampicin^48^). A well-studied member of this groups is epigallocatechin gallate (EGCG), a polyphenol sharing the vicinal hydroxyls implicated in dopamine’s interaction with α-synuclein. Despite their chemical similarities, dopamine and EGCG have divergent effects on the structure of α-synuclein^49^. In fact, while dopamine may induce toxic oligomers, adding EGCG during cell-free α-synuclein aggregation produces species less toxic to cells^39^. Other aggregation inhibitors alter α-synuclein-protein or -lipid interactions to prevent aggregation (e.g. NPT100-18A^50^, squalamine^51^, PcTS^52,53^, Hsp70^54^). Recent studies have examined small molecules that directly stabilize α-synuclein monomers (e.g. BIOD303^55^, nortriptyline^56^, CLR01^57,58^). However, it remains unknown whether these small molecules perturb α-synuclein’s lipid interactions, which are directly implicated in its role in neurotransmitter release^59–61^.

In this study we employed nordihydroguaiaretic acid (NDGA), a phenolic dibenzenediol lignan that inhibits α-synuclein aggregation, to examine the mechanisms of α-synuclein aggregation inhibitors and evaluate its potential utility as a chemical platform for the development of novel aggregation inhibitors^40,62^. Recent work has exhaustively characterized the products and kinetics of NDGA oxidation as well as several novel analogs NDGA and several novel analogs^63^. We provide evidence that cyclized NDGA, formed during oxidation, interacts with α-synuclein to produce modified monomers. We demonstrate that these aggregation-resistant monomers retain their capacity to interact with phospholipid membranes and that they inhibit aggregation of untreated α-synuclein. Further, we demonstrate for the first time that cyclized NDGA reduces α-synuclein-driven neurodegeneration in a relevant animal model. Generating α-synuclein species that retain native structural dynamics while exerting a dominant-negative effect on α-synuclein aggregation and neurotoxicity represents a new paradigm for intervening in PD and related disorders.

## Results

### NDGA inhibits both primary and secondary nucleation-mediated α-synuclein aggregation

**Figure 1:**
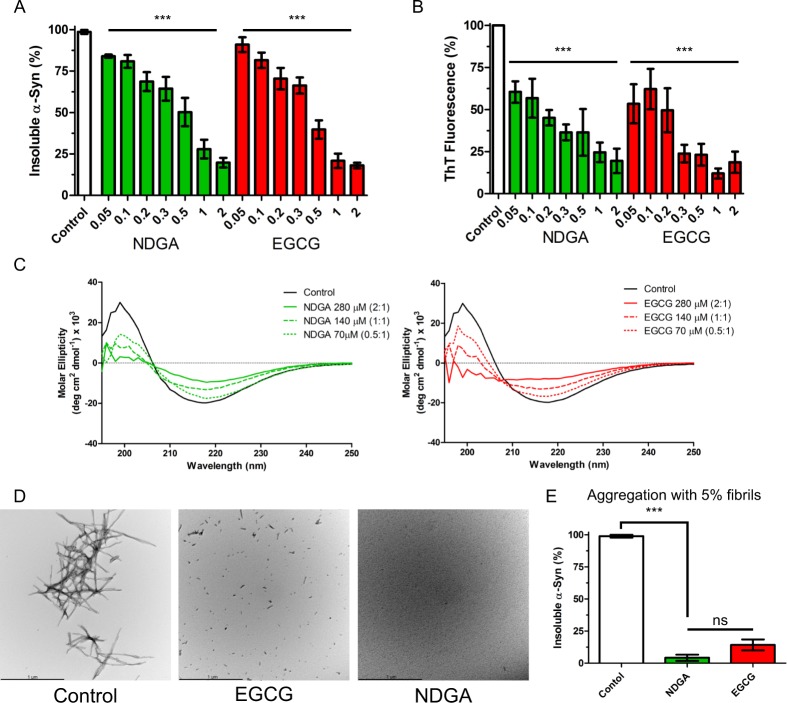
NDGA inhibits recombinant human α-synuclein aggregation. (**A**) Insoluble α-synuclein present after 7 days aggregation was reduced by NDGA and EGCG in a dose-dependent fashion as compared to solvent control. Recombinant human wildtype α-synuclein (138 µM) was aggregated for 7 days in the presence of EGCG or NDGA at the indicated molar ratios. After aggregation, PBS-insoluble α-synuclein was separated by centrifugation (21k g for 10 min). Soluble and insoluble fractions were boiled in SDS, run by SDS-PAGE, and colloidal stained. α-Synuclein in each fraction was quantified by in-gel densitometry (n = 3–5, ***p < 0.001). (**B**) Formation of amyloid α-synuclein fibrils quantified by Thioflavin-T was reduced by NDGA and EGCG in a dose-dependent fashion as compared to control after 7 days aggregation (n = 3–5, ***p < 0.001). (**C**) α-Synuclein beta-sheet secondary structure was reduced by EGCG and NDGA in a dose dependent fashion. Recombinant human wildtype α-synuclein (138 µM) was aggregated for 7 days in the presence of EGCG or NDGA at the indicated molar ratios. Secondary structure was quantified by circular dichroism. (**D**) Transmission electron microscopy images of α-synuclein aggregates after 3 days aggregation with small molecules at 1:1 molar ratio. (**E**) Insoluble α-synuclein present after 7 days aggregation in the presence of a 5% fibril seed was reduced by EGCG and NDGA. EGCG or NDGA were present at a 1:1 molar ratio. After aggregation, PBS-insoluble α-synuclein was separated by centrifugation (21k g for 10 min). Soluble and insoluble fractions were boiled in SDS, run by SDS-PAGE, and colloidal stained. α-Synuclein in each fraction was quantified by in-gel densitometry (n = 3, ***p < 0.001).

Two established assays, measurement of insoluble protein and Thioflavin-T binding, quantified the effect of NDGA on the aggregation of monomeric α-synuclein. NDGA caused concentration-dependent inhibition of insoluble α-synuclein accumulation and amyloid-like aggregate formation (Figs 1A,B and S1). Maximal inhibition was achieved at stoichiometric, equimolar concentrations of NDGA to monomeric α-synuclein. To confirm the Thioflavin-T findings, the secondary structure of aggregates was quantified by circular dichroism (CD). NDGA caused a dose-dependent decrease in the β-sheet content of the α-synuclein aggregates, as indicated by molar ellipticity decreases at 200 nm and increases at 220 nm (Fig. 1C). Finally, transmission electron microscopy was used to image the α-synuclein aggregates produced in the presence or absence of NDGA. Typical α-synuclein fibrils were observed in the absence of NDGA, but not after aggregation with equimolar NDGA (Fig. 1D). These data demonstrate that stoichiometric concentrations of NDGA inhibit the formation of amyloid-like α-synuclein fibrils.

The preceding experiments examined the effect of NDGA on a typical α-synuclein aggregation assay, in which primary nucleation is the rate-limiting step for the formation of fibrils. Aggregation of α-synuclein can also proceed by a secondary nucleation surface-catalyzed formation of fibrils. To test the effect of NDGA on secondary nucleation-dependent aggregation, α-synuclein fibrils (representing 5% of total α-synuclein) were added with monomeric α-synuclein. Equimolar NDGA (molecule to monomeric α-synuclein) inhibited the formation of insoluble α-synuclein aggregates in the presence of fibril seeds (Fig. 1E). Together, these findings demonstrate that stoichiometric NDGA prevents both primary and secondary nucleation dependent aggregation of α-synuclein.

### Interaction with α-synuclein requires oxidation and cyclization of NDGA

**Figure 2:**
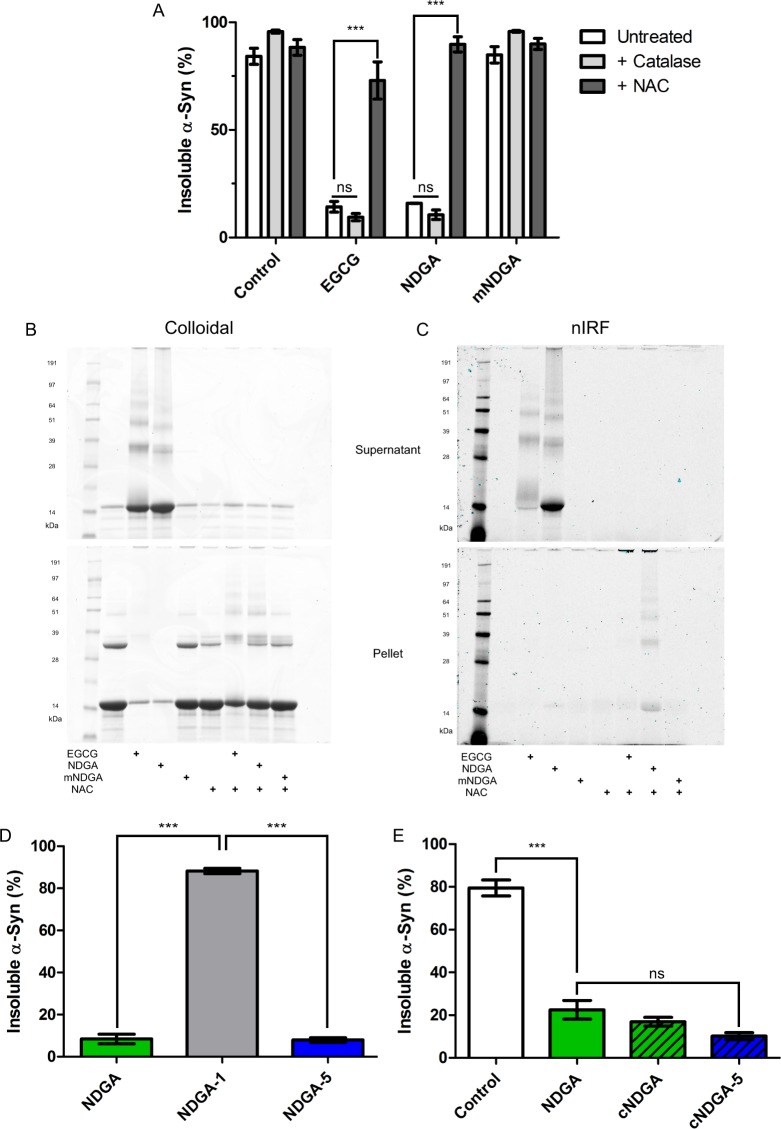
Interaction between NDGA and α-synuclein requires NDGA oxidation and cyclization. (**A**) NDGA treatment did not affect α-synuclein aggregation in the presence of N-acetylcysteine (NAC). α-Synuclein was aggregated for 3 days in the presence of 1:1 small molecules. After aggregation, PBS-insoluble α-synuclein was separated by centrifugation (21k g for 10 min). Soluble and insoluble fractions were boiled in SDS, run by SDS-PAGE, and colloidal stained. α-Synuclein in each fraction was quantified by in-gel densitometry. NAC was added at 20x molar excess to small molecule and catalase was added equal to 5% of protein, providing excess hydrogen peroxide decomposition capacity (n = 3–5, ***p < 0.001). (**B**) Colloidal staining of representative gels showed the formation of insoluble α-synuclein aggregates in the presence of NDGA and NAC. (**C**) Near-infrared fluorescent imaging of the same gels before colloidal staining showed a reduction of quinone-dependent fluorescence in the presence of NAC. (**D**) α-Synuclein did not aggregate in the presence of cyclizable analogs, NDGA and NDGA-5, but did with non-cyclizable NDGA-1 (n = 3, ***p < 0.001). (**E**) α-synuclein did not aggregate in the presence of cyclized cNDGA and cNDGA-5 (n = 3). α-Synuclein was aggregated for 3 days in the presence of 1:1 small molecules. After aggregation, PBS-insoluble α-synuclein was separated by centrifugation (21k g for 10 min). Soluble and insoluble fractions were boiled in SDS, run by SDS-PAGE, and colloidal stained. α-Synuclein in each fraction was quantified by in-gel densitometry.

We used N-acetylcysteine (NAC), an electron donor, to inhibit NDGA oxidation in the presence of α-synuclein. NAC was included in a typical α-synuclein aggregation mixture at 20:1 molar excess to NDGA. After aggregation for 3 days, levels of α-synuclein aggregation were measured by the established solubility assay. mNDGA, a NDGA analog incapable of oxidation and cyclization was included as a negative control (Supplementary Fig. S2). The addition of NAC allowed α-synuclein to aggregate in the presence of NDGA, suggesting that oxidation is necessary for NDGA to inhibit aggregation (Fig. 2A,B). The inclusion of NAC also reduced levels of quinone-containing NDGA oxidation products detected by near-infrared fluorescence (nIRF), a technique developed to study the oxidation-dependent interaction between α-synuclein and dopamine^64^ (Fig. 2C). The correlation of increased aggregation and decreased quinone nIRF suggests a relationship between NDGA oxidation and inhibition of α-synuclein aggregation. Separately, catalase, which rapidly converts H_2_O_2_ to water and hydrogen gas, was included in the aggregation assay to determine whether H_2_O_2_ that might be produced during NDGA oxidation mediated the effects on aggregation. Inclusion of catalase had no effect on α-synuclein aggregation in the presence of NDGA nor on quinone nIRF, indicating that the effect is not H_2_O_2_-dependent (Figs 2A and S3). These data indicate that inhibition of α-synuclein aggregation by NDGA and formation of quinone-modified α-synuclein is oxidation-dependent but not mediated by peroxide chemistry.

Previous work has identified the majority product of NDGA oxidation as a cyclized form^63^. During that study, novel NDGA analogs with defined oxidation pathways were generated. Here, these analogs were used to examine of the role of cyclization in NDGA’s oxidation-dependent inhibition of α-synuclein aggregation. We employed NDGA and two such analogs: NDGA-1, which oxidizes with comparable kinetics but does not cyclize due to unilateral hydroxyl substitution, and NDGA-5, which cyclizes more readily than NDGA due to reduced steric hindrance in the crosslinking region (Supplementary Fig. S2). α-Synuclein was aggregated for 3 days in the presence of equimolar NDGA analogs, and aggregation was quantified by the solubility assay. Both cyclizing analogs, NDGA and NDGA-5, inhibited α-synuclein aggregation, while non-cyclizing NDGA-1 did not (Fig. 2D). The products of the above experiment where also analyzed by nIRF. NDGA and NDGA-5-treated α-synuclein exhibited quinone nIRF signals, while NDGA-1-treated α-synuclein did not (Supplementary Fig. S4). These results indicate that cyclized forms of NDGA, or the process of cyclization, are required for inhibition of α-synuclein aggregation and formation of quinone-modified α-synuclein. To address this uncertainty, α-synuclein was aggregated in the presence of cNDGA and cNDGA-5, the purified cyclized forms of NDGA and NDGA-5, respectively (Supplementary Fig. S2). Using the solubility assay, both cNDGA and cNDGA-5 inhibited α-synuclein aggregation as potently as NDGA (Figs 2E and S5). This indicates that NDGA’s inhibition of α-synuclein aggregation is mediated by interaction of the protein with cyclized oxidation products.

### NDGA induces modest, progressive α-synuclein compaction without preventing dynamic adoption of α-helical conformation

During analysis of the interaction between NDGA and α-synuclein, equimolar incubation consistently produced nIRF-positive quinone-modified α-synuclein (Supplementary Fig. S6). Based on this observation, NDGA-treated α-synuclein was further analyzed to determine any structural effects of the interaction. Native nano-electrospray ionization mass spectra (ESI-MS), collected following 10 minute incubations of the protein-ligand mixtures, detected α-synuclein-NDGA complexes with masses matching the theoretical values of the unmodified protein and ligand^65^. This result indicates that the initial interaction between α-synuclein and NDGA does not require, nor produce, covalent modifications. Complexes were observed with higher relative intensities at low protein charge states, suggesting a preferential binding to the more compact α-synuclein conformations as previously observed with EGCG. However, levels of α-synuclein binding were much lower for NDGA than previously observed with EGCG^49^ (Supplementary Fig. S7).

**Figure 3:**
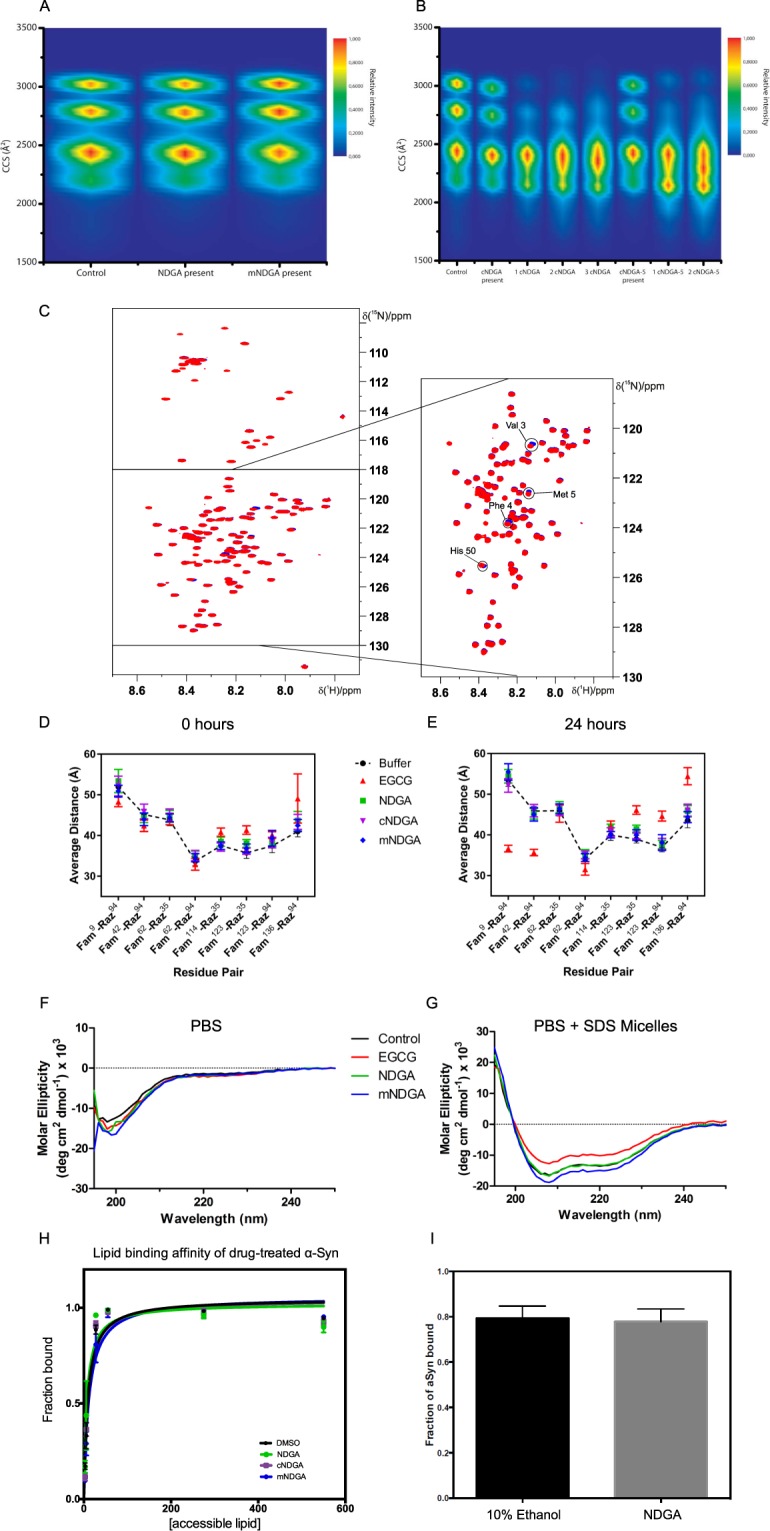
NDGA induces compaction of α-synuclein without preventing structural remodeling. (**A**) NDGA and mNDGA treatment did not alter α-synuclein collisional cross section measured by IM-MS. (**B**) cNDGA and cNDGA-5 induced α-synuclein compaction as measured by IM-MS. α-Synuclein was incubated with each molecule at 5:1 molar excess for 10 minutes before measurement. (**C**) NDGA treatment of α-synuclein did not induce extensive shifts in 2D NMR spectra. α-Synuclein was incubated 1:1 with NDGA for 24 hours before spectra were collected. NDGA-treated α-synuclein spectra (blue) was overlaid on solvent-treated α-synuclein (red). Fluorescein-maleimide (Fam) and tetramethylrhodamide azide (Raz) residue FRET measurement of (**D**) 0 hour and (**E**) 24 hour small molecule treatments showed progressive alteration of α-synuclein intramolecular distances by EGCG, but not NDGA, cNDGA, or mNDGA. The dashed line depicts 1 µM α-synuclein treated with buffer (1x PBS). Treatments were 5 µM NDGA (green square), EGCG (red triangle), cNDGA (purple inverted triangle), and mNDGA (blue diamond) (n = 3). (**F**) α-Synuclein secondary structure was not altered by pretreatment with NDGA. **(G)** NDGA pretreatment did not prevent α-synuclein assuming α-helical secondary structure in the presence of SDS micelles. α-Synuclein was incubated 1:1 with NDGA, mNDGA, or solvent alone for 24 hours and then dialyzed against PBS for 24 hours. 40 mM SDS micelles were added 5 minutes before analysis. Secondary structure was quantified by circular dichroism. (**H**) NDGA treatment did not alter α-synuclein phospholipid binding. α-Synuclein was incubated 1:1 with NDGA analogs or solvent alone for 24 hours before fluorescence correlation spectroscopy in the presence of POPS:POPC vesicles at the indicated concentrations (n = 3). (**I**) Addition of NDGA did not displace fluorescently labeled α-synuclein from POPS:POPC vesicles (n = 3).

Ion-mobility mass spectrometry (IM-MS) allows for highly sensitive detection of changes in ion compactness, mass, and charge. Of note, this allows subtle changes in protein conformation e.g. caused by ligand binding to alternative sites, to be distinguished by compactness even at the same mass and charge of the ion. Further, native IM-MS can resolve multiple different binding events by their conformational effects without averaging across the species distribution. This capability has previously been employed to show the differential effects of EGCG and dopamine on α-synuclein compaction^49^. IM-MS revealed that α-synuclein compaction was unchanged by incubation with either NDGA or mNDGA (Figs 3A and S7). However, incubation with cyclized NDGA analogs cNDGA and cNDGA-5 led to α-synuclein assuming more compact conformations (Fig. 3B). α-Synuclein did non-covalently bind cyclized analogs up to 3 times, as seen by the corresponding mass shifts, and further, interaction with greater numbers of cyclized analogs correlated with increased compaction. This finding recapitulates similar results after α-synuclein incubation with EGCG^49^ and indicates that interaction with cyclized NDGA causes structural compaction of α-synuclein.

NMR allowed for localization of NDGA’s structural effects within the sequence of α-synuclein. After 24 hours incubation with 1:1 NDGA small variations in peak positions were observed in the N-terminus – Val 3, Phe 4, Met 5 – as well as His 50 (Fig. 3C). cNGDA and NDGA-1-treatment caused similar changes in the spectra (data not shown). Incubation with 3:1 NDGA resulted in more pronounced shifts in the same set of peaks (Supplementary Fig. S8).

These samples were followed for several days through NMR revealing a progressive variation of the peak shifts. After several days, additional changes occurred in the signals of several peaks. These peaks constitute a second set of signals, indicative of a subset of the α-synuclein population with altered conformation at these residues.

Förster resonance energy transfer (FRET) analysis of α-synuclein provided further insight into the regions altered by NDGA treatment. This examination employed several synthetic α-synuclein proteins with varying fluorescent label pairs spread throughout the amino acid sequence. Based on this methodology, FRET signal from an individual label pair can be converted to an intramolecular distance within α-synuclein and, when taken together, the multiple label pairs provided eight partially overlapped measurements covering the amino acid sequence from residue 9 to 136^66^. FRET revealed progressive changes in α-synuclein conformation when treated with EGCG, while NDGA, cNDGA, and mNDGA did not induce changes, even after 24 hours (Fig. 3D,E). As with IM-MS and NMR, NDGA-treatment was found to induce minimal changes in α-synuclein conformation, while EGCG showed more marked effects.

NDGA-treated and dialyzed α-synuclein examined by CD showed no changes in secondary structure further confirming the IM-MS and NMR analyses (Fig. 3F). Despite observing limited effects of NDGA on α-synuclein structure in aqueous solution, we also examined whether NDGA treatment alters the capacity of α-synuclein to interact with hydrophobic membranes. Membrane interaction induces the N-terminus of α-synuclein to assume an α-helical secondary structure^67–69^. Capacity for membrane interaction is necessary for α-synuclein’s putative biological functions in the synapse^15,18,19^. Neither NDGA nor mNDGA treatment prevented α-synuclein from assuming α-helical conformations in the presence of SDS, just as occurred under control conditions (Fig. 3G). This indicates that the structural changes induced by NDGA do not restrict α-synuclein dynamic flexibility.

The ability of NDGA-treated α-synuclein to interact with membranes was further examined by fluorescence correlation spectroscopy (FCS), which has been used extensively to study the interaction of α-synuclein with lipid vesicles^16,70^. The binding of fluorescently labelled recombinant human α-synuclein to various concentrations of phospholipid vesicles (1:1, POPS:POPC) was compared following α-synuclein incubation for 24 hours with equimolar NDGA analogs or solvent alone. NDGA, cNDGA, and mNDGA did not alter phospholipid affinity as indicated by similar protein binding affinity at each vesicle concentration, regardless of treatment condition (Fig. 3H). FCS was also used to determine whether the addition of NDGA would displace α-synuclein already bound to phospholipid vesicles. The fraction of α-synuclein bound to vesicles was not altered by the addition of NDGA as compared to the addition of solvent alone (Fig. 3I). These data further indicate that NDGA treatment does not perturb α-synuclein interaction with hydrophobic membranes. Similarly, EGCG did not disrupt phospholipid or vesicle binding (Supplementary Fig. S9). Collectively, these results show that NDGA causes α-synuclein compaction across the sequence, without altering secondary structure or preventing structural remodeling involved in physiological function.

### NGDA pretreated α-synuclein stably resists seeded aggregation

**Figure 4:**
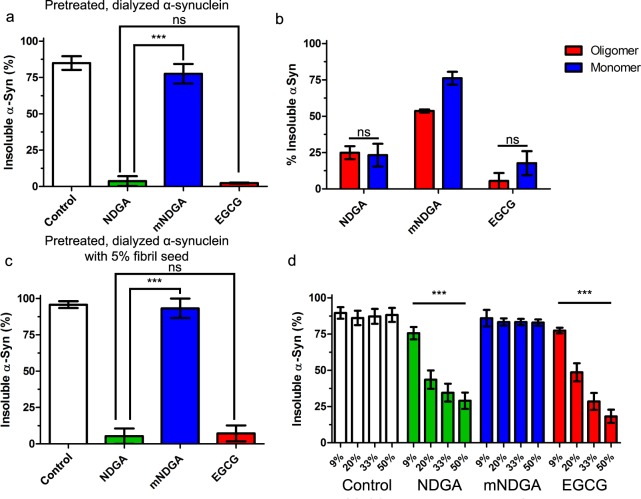
NDGA pretreatment prevents α-synuclein aggregation. (**a**) α-Synuclein did not aggregate after pretreatment with NDGA. α-Synuclein was incubated 1:1 with small molecules for 24 hours then dialyzed against PBS for 24 hours. After aggregation for 3 days, PBS-insoluble α-synuclein was separated by centrifugation (21k g for 10 min). Soluble and insoluble fractions were boiled in SDS, run by SDS-PAGE, and colloidal stained. α-Synuclein in each fraction was quantified by in-gel densitometry (n = 3, ***p < 0.001). (**b**) Both oligomeric and monomeric α-synuclein species induced by NDGA treatment resist aggregation. α-Synuclein was incubated 1:1 with small molecules for 24 hours then subjected to native state size exclusion chromatography. Oligomeric (≥51 Å) and Monomeric (<51 Å) α-synuclein fractions were collected and aggregated separately for 3 days. Soluble and insoluble species were separated and quantified as above (n = 3). (**c**) NDGA-treated α-synuclein resisted fibrillization in the presence of 5% fibril seed. α-Synuclein was incubated 1:1 with small molecules for 24 hours then dialyzed against excess PBS for 24 hours. Untreated α-synuclein fibrils equal to 5% total protein was added immediately before mixtures were aggregated for 3 days. Soluble and insoluble species were separated and quantified as above (n = 3, ***p < 0.001). (**d**) NDGA pretreated, dialyzed α-synuclein inhibited aggregation of untreated α-synuclein. α-Synuclein was incubated 1:1 with small molecules for 24 hours then dialyzed against excess PBS for 24 hours. Pretreated, dialyzed α-synuclein was then mixed with untreated monomeric α-synuclein at the indicated ratios. Mixtures were aggregated for 3 days. Soluble and insoluble species were separated and quantified as above (n = 3, ***p < 0.001).

Based on the progressive remodeling of α-synuclein treated with NDGA, we analyzed the capacity of NDGA-pretreated α-synuclein to aggregate into fibrils. α-Synuclein was incubated with equimolar NDGA (as well as mNDGA and EGCG controls) for 24 hours before dialysis against excess PBS for 24 hours. This pretreated, dialyzed α-synuclein was then aggregated for 3 days before undergoing the solubility assay (as depicted in Supplementary Fig. S10). NDGA, but not mNDGA, pretreatment prevented α-synuclein aggregation (Fig. 4A). Further, pretreated, dialyzed α-synuclein was aggregated for 14 days to test the duration of the effect. Again, NDGA but not mNDGA pretreatment prevented α-synuclein aggregation (Supplementary Fig. S11). Consistent with previous data, we found that pretreatment with cyclizing (NDGA and NDGA-5) and cyclized (cNDGA and cNDGA-5) analogs prevented α-synuclein aggregation, while non-cyclizing analogs (NDGA-1 and SECO-1) did not (Supplementary Figs S12 and S13). We also examined the soluble species that remained following aggregation of pretreated, dialyzed α-synuclein using native state SEC and found that monomeric species are the majority product (Supplementary Fig. S14). Collectively, these findings demonstrate the NDGA pretreatment renders α-synuclein monomers resistant to aggregation. This effect was not observed with non-oxidizable and non-cyclizing analogs of NDGA.

Native-state size exclusion chromatographic (SEC) fractionation found that pretreatment with either NDGA or EGCG produces both monomeric and oligomeric α-synuclein species (Supplementary Fig. S6). We compared the aggregation of these two pools of α-synuclein species. α-Synuclein was incubated with equimolar concentrations of small molecules for 24 hours before oligomeric (≥51 Å) and monomeric (<51 Å) species were separated using native state SEC. The resulting fractions were then aggregated for 3 days before analysis by the solubility assay. Both monomers and oligomers produced upon NDGA and EGCG pretreatment resisted aggregation, while the majority of mNDGA treatment products formed insoluble aggregates (Fig. 4b). This indicates that while aggregation-resistant α-synuclein oligomers do form after NDGA treatment, resulting monomers are also aggregation-resistant, even in the absence of oligomers.

Next, we explored whether NDGA can prevent surface-catalyzed, secondary nucleation-dependent, aggregation of α-synuclein. Pretreated, dialyzed α-synuclein was aggregated for 3 days in the presence of α-synuclein fibril seeds (equal to 5% of total α-synuclein in solution) before aggregation was quantified by the solubility assay. Again, NDGA but not mNDGA pretreatment reduced α-synuclein aggregation (Fig. 4c). This demonstrates that NDGA-pretreated, dialyzed α-synuclein does not readily undergo secondary nucleation to form species capable of incorporating into existing fibrils.

Based on the observation that NDGA treatment prevents α-synuclein aggregation, we conducted a novel analysis to determine whether aggregation of untreated α-synuclein is altered by the presence of NDGA-pretreated α-synuclein. Pretreated, dialyzed α-synuclein was generated as before, then mixed with untreated α-synuclein at varying ratios and the resulting mixtures were aggregated for 3 days before undergoing the solubility assay (as depicted in Supplementary Fig. S15). The addition of pretreated, dialyzed α-synuclein caused a super-stoichiometric reduction in insoluble α-synuclein, demonstrating that aggregation of untreated α-synuclein was reduced (Fig. 4d). This effect is demonstrated in the 20% condition wherein NDGA pretreated, dialyzed α-synuclein represents only 20% of total α-synuclein, but insoluble α-synuclein is reduced from 88% in control conditions to 37%. These data, taken in totality, indicate that pretreatment with NDGA produces α-synuclein monomers that are not only resistant to aggregation, but reduce aggregation of α-synuclein never exposed to NDGA.

### cNDGA reduces α-synuclein-driven dopamine neurodegeneration in animals

**Figure 5:**
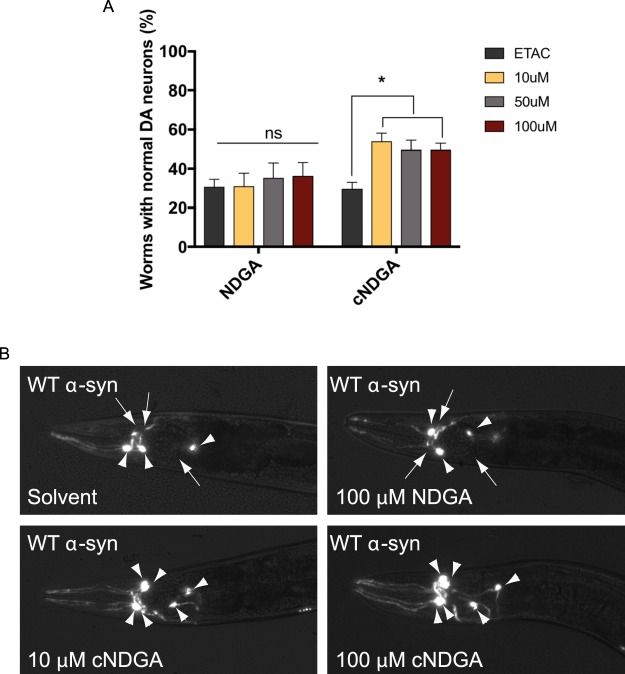
cNDGA reduces α-synuclein-driven neurodegeneration (**A**) cNDGA, but not NDGA reduces dopaminergic neurodegeneration in *C. elegans* expressing wildtype α-synuclein. Animals were exposed to each concentration of the drugs on days 0–3, 5, and 7 post-hatching. Animals were scored on day 8 post-hatching for dopaminergic neurodegeneration. The data are represented as mean ± SEM; one-way ANOVA with Tukey’s *post hoc* test for multiple comparisons (n = 3; 30 animals per replicate; *p < 0.05; ns, not significant). (**B**) Representative images from day 8 post-hatching. Arrowheads indicate intact neurons while arrows indicate degenerating or missing neurons. Scale bar represents 10 µm.

We examined the effect of NDGA and cNDGA treatment on α-synuclein-driven neurodegeneration (Fig. 5A,B). We employed a widely used, *Caenorhabditis elegans (C. elegans)* model in which expression of human wildtype α-synuclein in dopaminergic neurons leads to progressive neurodegeneration^71^. Animals were treated with varying doses of NDGA, or cNDGA from hatching until the day before scoring on either day 6 or day 8. Animals were considered to be undergoing neurodegeneration if any dopamine neurons were absent or dendritic processes showed signs of dysfunction such as blebbing^72^. Representative images of animals scored during this experiment show the loss of fluorescent dopamine neuron processes and bodies in solvent and NDGA-treated animals (Fig. 5B). No differences were observed in NDGA or cNDGA treated animal after day 6 (data not shown). cNDGA at all concentrations (10, 50 and 100 µM) significantly reduced the number of animals undergoing dopamine neuron degeneration at day 8 (Fig. 5A). Similarly, EGCG (50 µM) protected against neuron loss (Supplementary Fig. S16). In this model, expression of α-synuclein was limited to just six neurons with each animal. As such, total α-synuclein levels were so low as to preclude biochemical analysis of the interaction between α-synuclein and NDGA or cNDGA. Likewise, we were unable to conduct pharmacokinetic analysis of our target neurons. Despite these limited reservations, these results demonstrate, for the first time, that cNDGA can reduce neurodegeneration caused by α-synuclein.

## Discussion

Because of the engagement in several neurodegenerative diseases, α-synuclein aggregation remains a promising target for therapeutic development. Using NDGA and novel analogs, we uncovered that NDGA oxidation and cyclization was required for formation of quinone-modified monomeric α-synuclein. This interaction caused slight compaction of the α-synuclein molecule but did not induce any noticeable changes in its intrinsically disordered state. Furthermore, NDGA-treated α-synuclein retained its ability to undergo conformational changes required for interaction with the membranes, and NDGA did not dissociate protein-membrane complexes. However, interaction of α-synuclein with NDGA and its cyclizing and cyclized analogues efficiently prevented α-synuclein aggregation into fibrils. Conversely, non-cyclizing NDGA analogs had no effect on fibril formation, ensuring the aggregation resistant effects of NDGA and its analogs is not an artifact of reduced contact between α-synuclein molecules. Indeed, NDGA-treated monomers not only resisted aggregation, but prevented aggregation of naïve untreated α-synuclein. Treatment with cNDGA reduced neurodegeneration in an animal model of α-synuclein neurotoxicity. Taken together, these findings suggest that NDGA and related molecules that prevent α-synuclein aggregation without perturbing its native intrinsic disorder, or associations, may be promising targets for PD and related disorders characterized by α-synuclein aggregation.

Attempts to develop therapeutic strategies targeting α-synuclein have included decreasing levels by altering expression^73^ and clearance^74,75^, immunological targeting^76,77^, and altering aggregation. Many aggregation inhibitors were identified through chemical library screens before being subjected to mechanistic analysis^32,40,41^. The neurotransmitter dopamine, and molecules that share structural and chemical similarities, were the first inhibitors identified and are among the most potent inhibitors of α-synuclein aggregation^32^.

Identification of dopamine and related phenols as inhibitors of α-synuclein aggregation has led to many studies of phenolic compounds as potentially neuroprotective inhibitors. The most studied of these is EGCG, which is currently undergoing clinical trials in multiple system atrophy, a synucleinopathy characterized by microglial inclusions^78^. EGCG was found to be neuroprotective in animals treated with MPTP (1-methyl-4-phenyl-1,2,3,6-tetrahydropyridine), which is a prodrug to the neurotoxin MPP^+^ (1-methyl-4-phenylpyridinium) that induces α-synuclein-dependent dopaminergic neurodegeneration^79–81^. Adding EGCG during *in vitro* α-synuclein aggregation results in generation of less toxic aggregated products^39^. Given the structural differences between EGCG and NDGA, as well as the existing knowledge about NDGA oxidation and the availability of analogs, we hope that studying NDGA as an α-synuclein aggregation inhibitor will provide new insight into the neuroprotective potential of inhibiting α-synuclein aggregation.

Our analysis revealed some substantial departures between the effects of NDGA and EGCG on α-synuclein. Konijnenberg *et al*. found, using IM-MS, that EGCG induces α-synuclein compaction^49^, while analysis conducted by the same group for this study showed that cNDGA and cNDGA-5 induce α-synuclein compaction (Fig. 3A,B). NDGA, however, did not induce α-synuclein structural change. Interestingly, FRET found that EGCG causes substantial α-synuclein remodeling, while NDGA and cNDGA have no measurable effect on intramolecular distances (Fig. 3C). We also observed consistent differences in the patterns of SDS-stable multimers present after α-synuclein was treated with EGCG and NDGA (Figs 2B and S3, S6, S14). This trend of subtle, but measurable, differences between the effect of the two molecules leaves open the possibility of different mechanisms of their interaction with α-synuclein and differing biophysical properties of the resulting species.

Previous studies of EGCG have focused on the oligomers formed when α-synuclein is aggregated in the presence of EGCG^39,82,83^. In this study, we found that α-synuclein aggregation in the presence of EGCG and NDGA does produce oligomers, but that monomers are the predominant product, representing more than 75% of soluble protein (Supplementary Fig. S14). Further, we found that α-synuclein monomers and oligomers induced by pretreatment with EGCG and NDGA both resisted aggregation (Fig. 4b). The corresponding aggregation experiments were conducted after applying size-exclusion chromatography, which removed from solution any EGCG or NDGA not interacting with α-synuclein, suggesting a stable interaction or modification of α-synuclein itself. Most strikingly, NDGA-pretreated species inhibited aggregation of untreated α-synuclein, suggesting a dominant-negative effect on aggregation. This effect, not previously described for EGCG or any other exogenous phenolic inhibitors, may contribute to their neuroprotective effects.

There are two proposed models of amyloid-like fibril formation and elongation, although the precise mechanism remains unclear. In the first model monomers form oligomers and oligomers then stack together to form fibrils. In the second model of fibril formation, oligomers are elongated to fibrils with the addition of monomers. Under either paradigm, the data indicate that NDGA or EGCG modified oligomers do not progress to insoluble species. Possibly due to the increased compaction, monomeric α-synuclein treated with NDGA analogs, when incorporated into oligomers or fibrils may prevent further elongation. Essentially, the addition of a NDGA analog-modified α-synuclein monomer will “cap” the aggregation intermediate preventing the further addition of either monomer or oligomer. The stoichiometry would indicate that under either model the incorporation of aggregation resistant NDGA or EGCG modified monomer into oligomers or fibrils is sufficient to prevent further aggregation. The combination of a durable modification of α-synuclein and a dominant negative effect on aggregation are very desirable, given concerns over polyphenol bioavailability in the brain^84^ and the hepatotoxic side effects seen with EGCG or NDGA^78,85^.

Lipid-bound α-synuclein is resistant to aggregation^86^, and appropriate membrane interactions are implicated in its physiological function^87^. As such, an ideal therapeutic targeting α-synuclein aggregation would allow normal lipid interaction. Interestingly, both CD and FCS showed that the membrane interactions of α-synuclein were not perturbed by NDGA-treatment (Fig. 3G–I). Preservation of α-synuclein membrane interaction may help explain the divergent effects of NDGA and dopamine on neuron viability. Indeed, α-synuclein oligomers induce membrane destabilization and dopamine-induced oligomers may contribute to neurodegeneration in synucleinopathies^36,37^. Further, the ability of α-synuclein to undergo native structural dynamics is necessary for formation of multimers implicated in physiological function^59,60^ and aggregation resistance^88,89^. Future studies aimed at identifying neuroprotective α-synuclein aggregation inhibitors would be well served by incorporating methods to determine whether membrane interactions and structural dynamics are maintained, as is the case with NDGA.

NDGA pretreatment induced an NMR peak shift at His 50 (Fig. 3C). His 50 is located within the second region that assumes an α-helix during α-synuclein interaction with curved lipid membranes, falling in sequence just after the fourth lysine-rich repeat^67,69^. His 50 also mediates an oligomer-stabilizing interaction between α-synuclein and divalent metal ions, particularly Cu(II)^90,91^. Additionally, His 50 to Gln mutation causes autosomal dominant familial PD and accelerates α-synuclein aggregation *in vitro*^92–94^. This suggests that interaction with His 50 could alter α-synuclein aggregation kinetics. Further investigation of the interaction between NDGA and His 50 might provide insight into NDGA’s inhibition of α-synuclein aggregation. Curiously, three other residues whose positions were perturbed by NDGA binding (Val 3, Phe 4, and Met 5) are located in the close proximity to the Asp 2, which is also engaged in Cu(II) binding^95^.

The chemistry underpinning dopamine and related small molecule modulators of α-synuclein aggregation includes the capacity for auto-oxidation and the presence of vicinal hydroxyl (catechol) moieties^33,35^. In this study, we have expanded the previous observations and documented many new mechanistic insights for the biochemistry of catechol structures to inhibit the aggregation of α-synuclein. We found that NDGA-analogs require two pairs of vicinal hydroxyls to interact with α-synuclein. NDGA-1, which contains only one pair of vicinal hydroxyl groups, did not modify α-synuclein or inhibit aggregation. This indicates that NDGA cyclization, which is enabled by its two pairs of vicinal hydroxyls, is required for inhibition of α-synuclein aggregation^63^. Indeed, cNDGA and cNDGA-5, two cyclized analogs of NDGA, were found to inhibit α-synuclein aggregation. The finding that NDGA’s cyclized oxidation products are responsible for its effects on α-synuclein aggregation presents the dual opportunities to study NDGA as a prodrug, with potential for conversion by oxidation-dependent cyclization to active cNDGA, and to examine novel molecules based on the cNDGA structure in hopes of identifying inhibitors of α-synuclein aggregation.

The importance of NDGA cyclization is reinforced by the finding that cNDGA, but not NDGA, treatment reduces neurodegeneration caused by expression of human α-synuclein in dopamine neurons in *C. elegans* (Fig. 5). There are many potential explanations for the divergent effects of the two molecules, including differences in stability, uptake, metabolism, and excretion. One possibility is that NDGA does not form cNDGA under these conditions. Oxidation of NDGA likely occurs less frequently in the reducing environment of the cell^96^, and yield of cNDGA may be lower than previously observed. While the cyclization of NDGA has been precisely described under controlled conditions in buffer^63^, it has never been examined in a complex milieu or within a living cell, where other chemistry may occur.

It could be of interest to compare the molecular mechanism of action of NDGA on α-synuclein with the effects of different small molecules on functionality of other intrinsically disordered proteins. One of the best studied examples of such systems is given by small molecule-driven inhibition of heterodimerization of the transcription factor c-Myc with its partner, Max, via a basic-helix-loop-helix-leucine zipper (bHLHZip) domain present in both proteins. Importantly, both proteins in their unbound forms are disordered, but undergo mutual coupled binding and folding when their zipper domains interact to form a helical coiled coil^97–99^. High throughput screening uncovered several specific inhibitors that were able to bind to one of three discrete sites (residues 366–375, 375–385, and 402–409) within the 85-residue bHLHZip domain of the monomeric c-Myc^100,101^. Solution NMR analysis revealed that interaction of said small molecules with c-Myc resulted in its local misfolding, thereby generating conformations incompatible with the heterodimerization of this protein with Max^97–99^. Clearly, the mechanism of NDGA action, where a small molecule stabilizes specific conformations of intrinsically disordered α-synuclein, is principally different from the inhibitory activity of small molecules inducing c-Myc misfolding. Therefore, further study is warranted to determine whether the interaction between NDGA and α-synuclein represents a new mode of modulatory action of small molecules on intrinsically disordered proteins. Moreover, in-cell NMR data indicated that molecular crowding agents present in the cytosol induce compaction of α-synuclein and shielding of the aggregation prone region (hydrophobic core between residues 61–95) preventing oligomerization and aggregation^102^. Although ensemble-based techniques (FRET or NMR) are not able to visualize and inform on the structures of these compact states, we speculate that the compact states observed by NDGA analogs and EGCG are similar to those observed with in-cell NMR.

Collectively, these findings change our understanding of the mechanisms of phenolic α-synuclein aggregation inhibitors. We demonstrate, for the first time, that inhibition of α-synuclein aggregation by NDGA requires oxidation-dependent cyclization, reframing NDGA as a prodrug. Additionally, we show that both NDGA and EGCG stably modify α-synuclein monomers, rendering them aggregation incompetent. In the case of NDGA, this is achieved without alteration of α-synuclein secondary structure in solution, nor perturbing membrane interactions. This combination of attributes has not been previously observed in other inhibitors of α-synuclein aggregation. Further examination is certainly warranted, including a full characterization of efficacy and critically toxicity, to determine whether NDGA analogs might provide the basis for novel neuroprotective therapies for PD and related synucleinopathies.

## Methods

### *In vitro* α-synuclein aggregation analyses

Recombinant human wild type α-synuclein was expressed and purified from *E. coli* as previously described^103^. Purified α-synuclein was used at 2 mg/ml (138 µM) with or without small molecules at various molar ratios in PBS with 1% DMSO. Mixtures were aggregated for 3 or 7 days at 37 °C shaking at 1400 rpm. The longer 7 day incubation was performed to ensure the treatment effects remained consistent even under prolonged aggregation. Alternatively, purified α-synuclein was incubated for 24 hours with or with equimolar small molecules in PBS with 1% DMSO. Resulting mixtures were then dialyzed against excess PBS for 24 hours at 4 °C (using mini-dialysis tubes, Thermo Fisher 69572). Mixtures were aggregated for 3 or 14 days shaking at 1400 rpm. Amyloid fibril formation was monitored by thioflavin T. Briefly, 4 µM protein was incubated with 25 µM ThT (Sigma Aldrich) dissolved in PBS and read at Ex of 450 and Em 482 using a fluorescent plate reader (Molecular Devices).

### α-Synuclein solubility analysis and densitometric quantification

Sedimentation was performed by centrifugation at 21,000 g for 15 minutes. Supernatants were removed, and pellets were resuspended in an equal volume. Pellets and supernatants were boiled in SDS sample buffer for 3 minutes at 95 °C. α-Synuclein species were resolved by SDS-PAGE in 12% Bis/Tris gels and stained in-gel with colloidal blue (Invitrogen LC6025). Stained gels were imaged at 700 nm using LI-COR Odyssey Infrared Imaging System and densitometric quantification was conducted using LI-COR Odyssey software suite 3.0.

### Near infrared fluorescence

SDS-PAGE gels were imaged as previously described^64^. Briefly, gels were imaged immediately after electrophoresis at 700 nm with intensity 10 on LI-COR Odyssey Infrared Imaging System.

### Size-exclusion chromatography

For size exclusion chromatography 200 µL recombinant α-synuclein (2 mg/mL) was injected onto a Superdex 200 10/300 GL (GE Healthcare) connected to an Agilent 1100 series HPLC system and fraction collector controlled by ChemStation software version 1.04 (Agilent). Mobile phase consisted of 25 mM HEPES and 150 mM NaCl, pH 7.25 with a 0.3 mL/min flow rate. Fractions corresponding to 140-100, 100-85, 85-75, 75-68, 68-61.5, 61.5-56, 56-51, 51-47, 47-43, 43-39, 39, 39-36, 36-32, 32-29, 29-26 Å were combined and concentrated using 3 kDa NMWL ultracel microcon filters (Millipore UFC5003). SEC column elution time was calibrated to Å size using globular protein standards (GE Healthcare).

### Circular dichroism

Circular dichroism spectra were obtained on a Jasco-810 spectropolarimeter maintained in the Children’s Hospital of Philadelphia Protein Core facility. Protein in PBS was diluted to 20 μM in 0.05 M KH_2_PO_4_ pH 7.8. Spectra were corrected for baseline measurement of an equivalent volume of PBS diluted in KH_2_PO_4_ buffer. Spectra were collected with a scanning width of 1 nm, at 5 nm/min with 2 accumulations per run.

### Transmission electron microscopy

Transmission electron microscopy was conducted on a JEOL1010 maintained in the University of Pennsylvania Electron Microscopy Resource Laboratory. Protein solutions were diluted to 0.5 mg/mL (34.5 µM) before mounting to carbon-coated 300 grids and negative staining with 2% uranium acetate. Images were collected with HV = 80.0 kV, Magnification 50,000x. Contrast was automatically adjusted during capture for each image, and not altered after capture. 1 µm scale bars were included for each image presented.

### Nuclear magnetic resonance spectroscopy

To provide atomic resolution information on α-synuclein, 2D ^1^H-^15^N NMR spectra were acquired through a series of band-selective excitation short-transient transverse relaxation optimized spectroscopy heteronuclear single quantum coherence (BEST-TROSY HSQC) runs^104^, a fast and common experiment used to evaluate if there is an effect of the different molecules on some amino acids, each of them represented by a H^N^-N correlation (a cross-peak in the 2D spectrum).

All the NMR experiments were recorded on a Bruker Avance III spectrometer operating at 900 MHz ^1^H frequency (21.14 T) equipped with a cryogenically cooled probehead for triple resonance experiments (TCI). The pulses involved in this pulse sequence were the standard ones used to investigate bio-molecules^105^. The ^15^N hard pulses were applied at the center of the region (117.02 ppm) and the ^1^H band selective pulses were applied at the center of the amide proton region at 8.70 ppm. A spectral width of 10822.511 Hz in the direct ^1^H dimension and 3647.991 Hz in the indirect ^15^N dimension was used. All the spectra were recorded with 8 scans per increment, 4096 and 2048 points in the direct dimension and in the indirect dimension respectively and a recycle delay of 500 ms.

The experiments were recorded at 288.0 K on a 165 μM uniformly ^15^N labeled α-synuclein sample, prepared as previously described^106^, in buffer (10 mM KPi, 140 mM NaCl, 0.25 mM EDTA + Roche protease inhibitors, pH 7.5). 5% D_2_O was added for the lock signal. The different drugs (described above) were added as DMSO solutions at 1:1 stoichiometric ratio and left at room temperature 24 hours to incubate before NMR measurements. The same experiments were acquired on a sample containing 3 equivalents of NDGA. Care was taken to minimize the volume of the DMSO solution added and to keep it constant for all the samples investigated. All the samples were analyzed again in the following days to evaluate thermodynamic and kinetic effects.

### Native nano-electrospray ionization mass spectrometry

ESI-MS experiments were conducted as previously described^49^. The only methodological departure was the inclusion of DMSO to solubilize the ligands. Briefly, MS spectra were collected after 10 minute incubation of protein-ligand mixtures in pH 7.4 10 mM ammonium acetate. Spectra were collected using a hybrid quadrupole-time-of-flight mass spectrometer (QSTAR-Elite, Biosystems, Foster City, CA) equipped with a nano-ESI sample source.

### Native Ion mobility mass spectrometry

Native Ion mobility-mass spectrometry (IM-MS) experiments were conducted as previously described^49^. The only methodological departure was the inclusion of DMSO to solubilize the ligands. Briefly, MS spectra were collected after 10 minute incubation of protein-ligand mixtures in pH 7.4 10 mM ammonium acetate. α-Synuclein was present in the mixtures at 20 µM, ligand at 100 µM. IM-MS was performed on a Synapt G2 HDMS (Waters, Manchester, U.K.) using nano-ESI with homemade gold-coated borosilicate capillaries.

### Fluorescence correlation spectroscopy

#### Expression of fluorescently-labeled α-synuclein

α-Synuclein protein labeled at residue S9C with Alexa Fluor 488 was produced via recombinant protein production. Plasmid containing α-synuclein S9C fused to a polyhistidine-tagged GyrA intein from Mycobacterium xenopi (Mxe) was transformed into BL21 DE3 competent cells by heat shocking at 42 °C. Single colonies grown on Ampicillin (Amp) plates were picked to inoculate primary cultures in LB supplemented with 1 μg/mL Amp. Primary cultures were combined into secondary cultures, which were grown at 37 °C in a shaker-incubator until optical density (OD) reached ~0.6. Expression of the gene of interest was induced with Isopropyl β-D-1-thiogalactopyranoside (IPTG). Cells were then grown in the shaker-incubator at 18 °C overnight. After centrifugation (5000 rpm, 20 min, 4 °C), cell pellets were re-suspended in re-suspension buffer (20 mM Tris, 1 mM PMSF, 1 Roche protease inhibitor tablet, pH 8.3) and sonicated in a cup in an ice bath (5 min, 1 s ON, 1 s OFF). The resulting lysate was centrifuged (14,000 rpm, 25 min, 4 °C), and supernatant containing the protein of interest (POI) was purified over Ni-NTA affinity column. Intein cleavage was carried out by incubation with 200 mM β-mercaptoethanol (βME) on a rotisserie over night at room temperature. Cleaved POI was dialyzed into 20 mM Tris pH 8 before purification over a second Ni-NTA column to remove the free intein from the sample. Flow-through containing the POI was kept, dialyzed into 20 mM Tris pH 8, and spin concentrated. Labeling with Alexa Fluor 488 (AF488) maleimide was done by adding 4 equivalents (eq.) of fluorophore dissolved in DMSO. The reaction tube was wrapped in aluminum foil and incubated at 37 °C for ~6 hrs. Formation of the product α-synuclein C9-AF488 was checked by matrix-assisted laser desorption ionization mass spectrometry (MALDI-MS) and polyacrylamide gel electrophoresis (SDS-PAGE). Labeled protein was purified by fast-protein liquid chromatography (FPLC) using a Hi-Trap Q 5 mL column and by reverse-phase high-performance liquid chromatography (RP-HPLC) using a C18 preparatory column. Purified protein was spin concentrated into buffer (20 mM Tris, 100 mM NaCl), and aliquots were stored at −80 °C.

#### Treating α-synuclein with drug molecules

The concentration of α-synuclein aliquots was determined by UV-Vis at 488 nm. The drug molecules NDGA, mNDGA, and cNDGA were dissolved in DMSO and diluted 10-fold into buffer (20 mM Tris, 100 mM NaCl). A control of 10% DMSO in buffer was also made. Each aliquot of α-synuclein was respectively treated with 1eq. of NDGA, mNDGA, and cNDGA, and DMSO-buffer. Treatment samples were incubated at room temperature for 8 hours, then dialyzed against 10 mM Tris, 100 mM NaCl overnight.

#### Preparation of lipid vesicles

Synthetic lipid vesicles were prepared for use in binding experiments. A mixture in 50:50 molar ratio of 1-palmitoyl-2-oleoyl-sn-glycero-3-phosphoserine (POPS) and 1-palmitoyl-2-oleoyl-sn-glycero-3-phosphocholine (POPC) were drawn from chloroform stock and dried under nitrogen gas to form a film inside a glass vial. Films were desiccated under vacuum and re-hydrated in 3-(N-morpholino)propanesulfonic acid (MOPS) buffer. 10 Freeze-thaw cycles consisting of cooling in liquid nitrogen for 40 s and warming in a 60 °C water bath for 2 min were performed to aid the formation of uniformly sized vesicles. Using syringes fitted onto an extruder, vesicles were pushed 31 times through 50 nm pore membranes. Vesicles were determined by Dynamic Light Scattering to be monodisperse and distributed uniformly around 80 nm in diameter, consistent across different concentrations of all samples. All lipid vesicles were prepared fresh and used within 48 hours of extrusion.

#### Fluorescence correlation spectroscopy

Eight-well chambered coverglasses (Nunc, Rochester, NY) were prepared by plasma cleaning followed by incubation over night with polylysine-conjugated polyethylene glycol (PEG-PLL), prepared using a modified Pierce PEGylation protocol (Pierce, Rockford, IL). PEG-PLL coated Chambers were rinsed with and stored in Milli-Q water until use. FCS measurements were done on a lab-built instrument based on an Olympus IX71 microscope with a continuous emission 488 nm DPSS 50 mW laser (Spectra-Physics, Santa Clara, CA). All measurements were done at 20 °C. The laser power entering the microscope was adjusted to 4.5 μW. Fluorescence emission collected through the objective was separated from the excitation signal through a Z488rdc long pass dichroic and an HQ600/200 m bandpass filter (Chroma, Bellows Falls, VT). Emission signal was focused through a 50 μm optical fiber. Signal was amplified by an avalanche photodiode (Perkin Elmer, Waltham, MA) coupled to the fiber. A digital autocorrelator (Flex03Q-12, correlator.com, Bridgewater, NJ) was used to collect 30 autocorrelation curves of 30 seconds for each measurement. Fitting was done using MATLAB (The MathWorks, Natick, MA).

#### Binding assay of drug-treated α-synuclein to lipid vesicles

FCS was used to examine the binding affinity of drug-treated α-synuclein to lipid vesicles. Each drug-treated α-synuclein labeled with AF488 was examined in the presence of varying concentrations of lipid vesicles, and each autocorrelation curve was fit to a 2-component equation to extract the fraction of bound α-syn at each concentration. From these data, a binding curve was generated and fit to an exponential to determine the dissociation constant Kd, i.e. the concentration at which half of the protein is bound. In fitting the autocorrelation curves for α-syn in the presence of lipid vesicles, the diffusion time (τ) of bound and unbound α-syn were respectively fixed to experimentally determined values. The diffusion time of unbound protein, ταS, was determined by measurements of the protein in buffer without lipids. Since bound protein diffuses with the vesicles to which they are bound, the diffusion time of the vesicles, τvesicle, was determined by measurements of BODIPY-labeled 50:50 POPS/POPC. The diffusion time of our unlabeled vesicles were also deduced from a calibration curve generated from the diffusion times of commercial fluorescent bead standards of 50 nm and 100 nm in diameter.

To generate a vesicle-binding curve, FCS was performed on α-syn C9-AF488 in the presence of varying concentrations (0.005 mM to 1 mM lipid) of 50:50 POPS/POPC. The accessible surface lipid concentration was calculated based on the characteristic bilayer thickness of POPS and POPC. The fraction of bound protein was extracted from the fit to each autocorrelation curve by fixing ταS and globally fitting τvesicle to the same value across all concentrations.

$$G(\tau )=\frac{1}{N}({F}_{F}\ast \frac{1}{1+\frac{\tau }{{\tau }_{\alpha S}}}\ast {(\frac{1}{1+\frac{{s}^{2}\tau }{{\tau }_{\alpha S}}})}^{1/2}+(1-{F}_{F})\ast \frac{1}{1+\frac{\tau }{{\tau }_{vesicle}}}\ast {(\frac{1}{1+\frac{{s}^{2}\tau }{{\tau }_{vesicle}}})}^{1/2})$$

The fraction bound was plotted against accessible lipid concentration and fit to an exponential, from which the Kd was determined.

$${F}_{B}=\frac{{B}_{{\max }}x}{{K}_{d,app}+x}$$

#### Assay for effect of NDGA on lipid-bound α-synuclein

Untreated α-synuclein C9-AF488 (final concentration 20 μM) was added to 250 μL of 0.05 mM POPS/PC in a microscope chamber well. The sample was incubated for 15 minutes to ensure maximum binding interactions. NDGA stock was made by dissolving the drug in ethanol and diluting 10-fold into buffer (20 mM Tris, 100 mM NaCl). NDGA was added at 20 μM final concentration to the vesicle-bound α-synuclein sample, and fraction of protein bound was determined using the fitting described above. A control treatment was made of 10% ethanol in buffer, and fraction of protein bound was determined in the same manner.

### Förster resonance energy transfer anaylsis

Small molecule stock solutions containing either NDGA, EGCG, cNDGA or mNDGA were prepared in DMSO. Labeled proteins constructs, including new constructs not previously described, were prepared using methods previously described^66^. Labeled positions not previously reported were confirmed by mass spectrometry of both the full-length protein and fragments from trypsin digestion using MALDI. The concentration of each protein stock was assessed by UV-Vis absorbance of the attached fluorescein-maleimide (Fam) (ε_494_ = 68,000 M^−1^ cm^−1^) and/or tetramethylrhodamide azide (Raz) (ε_555_ = 87,000 M^−1^ cm^−1^) dyes. Fluorescence assays were performed in a black, clear bottom half-diameter Greiner 96-well plate and measurements were taken on a Tecan M1000 plate reader. Each well of the plate contained a single protein sample, containing either one or both fluorescent labels, and one of the small molecules of interest. Protein samples were also measured in the absence of small molecules as a control. Each sample was prepared in 1X PBS with a final concentration of 1 μM protein. Samples containing NDGA, EGCG, cNDGA and mNDGA were prepared with a 5-fold excess of small molecules relative to the protein concentration. Small molecule stocks were prepared just prior to performing the assay. Each assay was performed by diluting the protein in buffer in each well and adding the small molecule solution, using a multichannel pipette, just prior to measuring the fluorescence. Each sample was excited at 494 nm and the emission spectrum was measured from 502–700 nm with a 1 nm step size. Each step consisted of 50 flashes at a frequency of 400 Hz with a 20 μs integration time and the gain was set to 135. The Z-position of the plate was optimized prior to the first measurement and was maintained at 21728 μm for all measurements. All measurements were performed at room temperature. After measuring the emission spectrum from each well immediately after the introduction of small molecule, the plates were sealed with parafilm and covered in aluminum foil and left at room temperature for 24 hrs. After 24 hrs the fluorescence of each sample was measured again as detailed above. The FRET between Fam and Raz probes attached to a single protein was assessed by computing the fluorescence quenching of the donor Fam fluorophore induced by the presence of the Raz acceptor as previously described^66^. FRET efficiencies were converted to average interresidue distances using the Förster equation and a gaussian chain polymer model.

### Analysis of dopaminergic neurodegeneration in *C. elegans*

#### Generation of transgenic nematodes

Transgenic *C. elegans* lines were generated by microinjection using previously described methods^107^. The strain UA294 (*baEx175 a,b,c* [P_*dat-1*_::WT a-syn, P_*unc-54*_::tdTomato]; *vtIs*7[P_*dat-1*_::GFP]) was generated by injecting with a solution of 50 ng/μL plasmid with either P_*dat-1*_::WT a-syn with a phenotypic marker (P_*unc-54*_::tdTomato, 50 ng/μL, for body wall muscle expression). Three distinct stable lines (*a, b, c*) were generated.

#### Analysis of dopaminergic neurodegeneration and NDGA analog treatments in *C. elegans*

NDGA and cNDGA were used at final concentrations of 10 µM, 50 µM, or 100 µM in ethyl acetate (EtAc). NDGA, cNDGA, or solvent (EtAc) was added to the surface of bacterial lawn on the nematode growth medium (NGM) Petri plates (60 mm diameter) at the corresponding final concentration and allowed to dry under the hood. For EGCG, 50 µM was added into the NGM agar plates. Three resulting independent transgenic lines (*a, b, c*) were synchronized, and exposed to corresponding concentration of NDGA, cNDGA, or solvent (EtAc) from hatching to day 3, then were transferred and maintained on NGM plates until the day of analysis. Animals on NGM plates were treated with additional NDGA, cNDGA, or solvent on days 5 and 7. For EGCG treatment, worms were transferred to fresh NGM plates daily and transferred onto fresh EGCG containing plates on days 0–3 and 5 post-hatching. For dopaminergic neurodegeneration analyses, the transgenic animals were scored as described previously^72^. Briefly, on the day of analysis, the six anterior dopaminergic neurons (four CEP (cephalic) and two ADE (anterior deirid)) were examined in 30 randomly selected nematodes with the marker transgene (tdTomato) in the body wall muscle cells. Worms were considered normal when all six anterior neurons were present without any signs of degeneration, such as broken dendritic process, cell body loss, dendritic blebbing, or a missing neuron. In total, at least 90 adult worms were analyzed for each treatment condition, at least 30 from each independent transgenic line. An average of total percentage of worms with normal neurons was reported in the study.

### Reagents

Nordihydroguaiaretic acid, NDGA 74540 Aldrich; (−)- Epigallocatechin gallate, EGCG E4143 Sigma or 4524 Tocris; Terameprocol/mNDGA, T3455 Sigma; Catalase, C9322 Sigma; NDGA analogs NDGA-1, SECO-1, NDGA-5, cNDGA, and cNDGA-5 were generated, purified, and validated by Krol *et al*. at the University of Saskatchewan as previously described^63^.

### Statistical analysis

Statistical analysis was conducted using Prism 5 software (GraphPad). Unpaired students *t* test was used for comparisons between two groups. Comparisons involving multiple groups, such as molecule and dose comparisons, used two-way ANOVA with Tukey’s *post hoc* test for multiple comparisons. In all cases *p* < 0.05 was considered statically significant.

## Supplementary information

Revised Supplement figures

## Data Availability

All data generated or analyzed during this study are included in this published article (and its Supplementary Information files).
