# Supplementary material for: Cyclized NDGA modifies dynamic α-synuclein monomers preventing aggregation and toxicity
Source: Sci Rep. 2019 Feb 27;9:2937. doi: 10.1038/s41598-019-39480-z (PMC6393491; doi:10.1038/s41598-019-39480-z)
Supplement: Supplementary file 1 — Revised Supplement figures [file 41598_2019_39480_MOESM1_ESM.docx]

**Supplementary Figures**

**Title:** Cyclized NDGA modifies dynamic α-synuclein monomers preventing aggregation and toxicity

**Authors:** Malcolm J. Daniels, J. Brucker Nourse Jr., Hanna Kim, Valerio Sainati, Marco Schiavina, Maria Grazia Murrali, Buyan Pan, John J. Ferrie, Conor M. Haney, Rani Moons, Neal S. Gould, Antonino Natalello, Rita Grandori, Frank Sobott, E. James Petersson, Elizabeth Rhoades, Roberta Pierattelli, Isabella Felli, Vladimir N. Uversky, Kim A. Caldwell, Guy A. Caldwell, Edward S. Krol and Harry Ischiropoulos


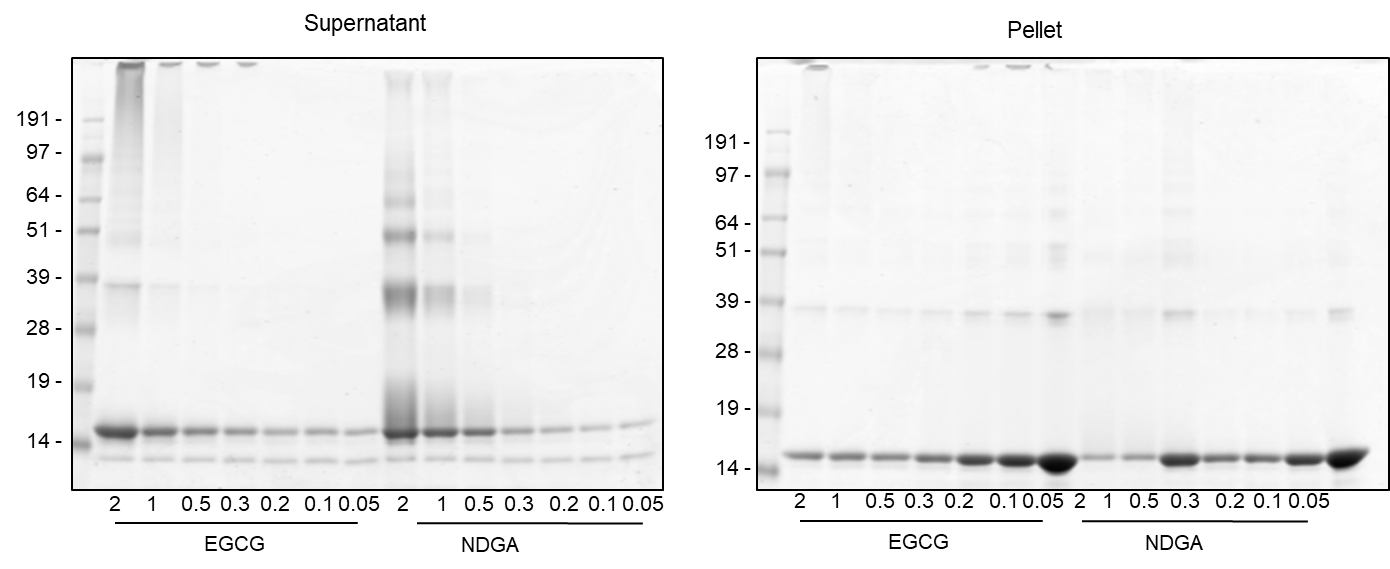


Supplementary Figure S1. Dose effect of EGCG or NDGA on α-synuclein aggregation. Representative images of recombinant human wildtype α-synuclein (138 µM) aggregated for 7 days in the presence of EGCG or NDGA at the indicated molar ratios. After aggregation, PBS-insoluble α-synuclein was separated by centrifugation (21k g for 10 min). Soluble (supernatant) and insoluble (pellet) fractions were boiled in SDS, run by SDS-PAGE, and colloidal stained. α-Synuclein in each fraction was quantified by in-gel densitometry. (n = 3-5).

Supplementary
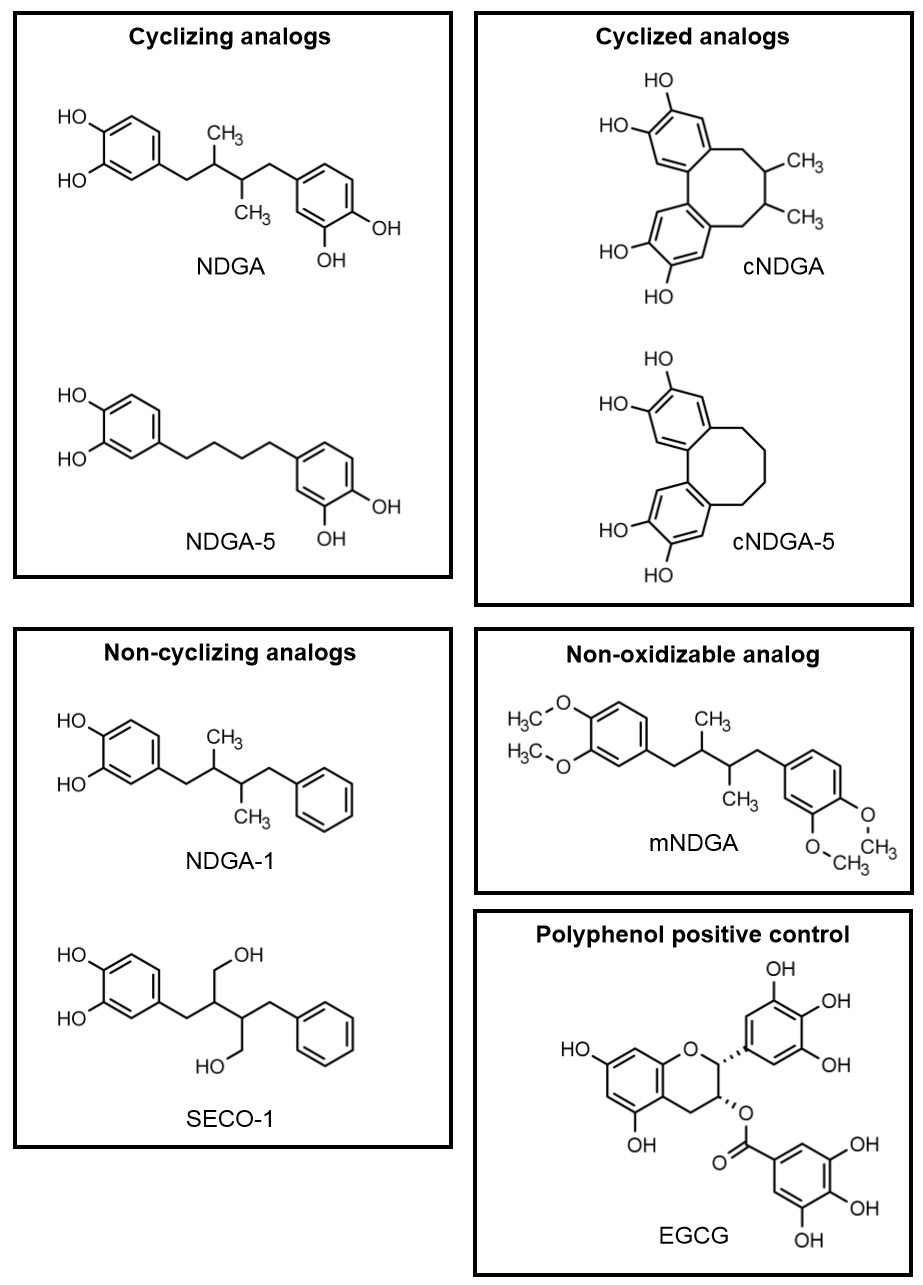
Figure S2. Structures of EGCG, NDGA, and NDGA analogs employed in this study.


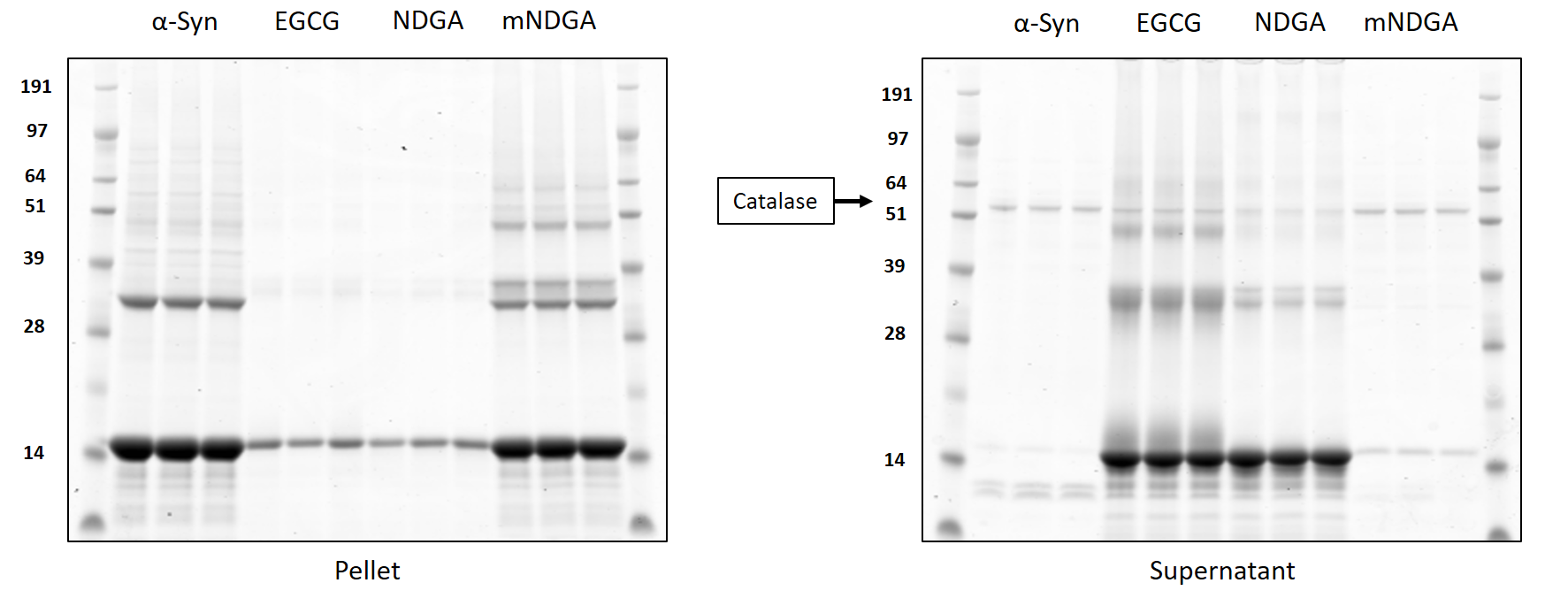


A

B


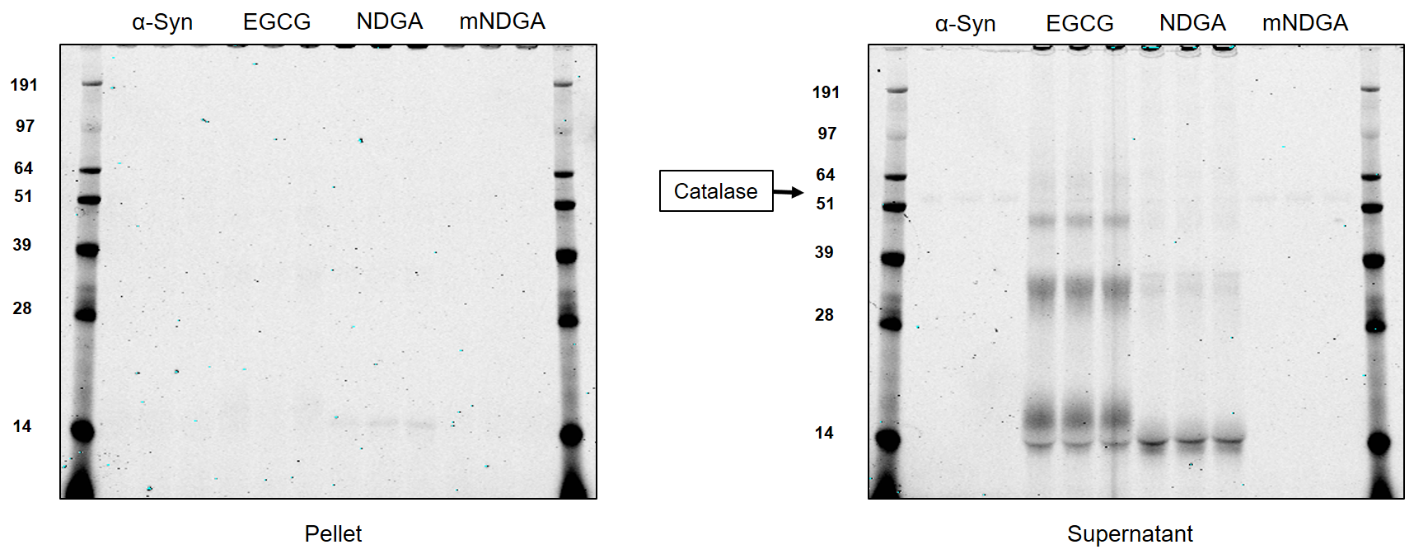


Supplementary Figure S3. Colloidal and nIRF images of α-synuclein aggregation in the presence of 5% catalase. **(a)** Colloidal stained gel **(b)** nIRF image of the same gel before colloidal staining, n=3.


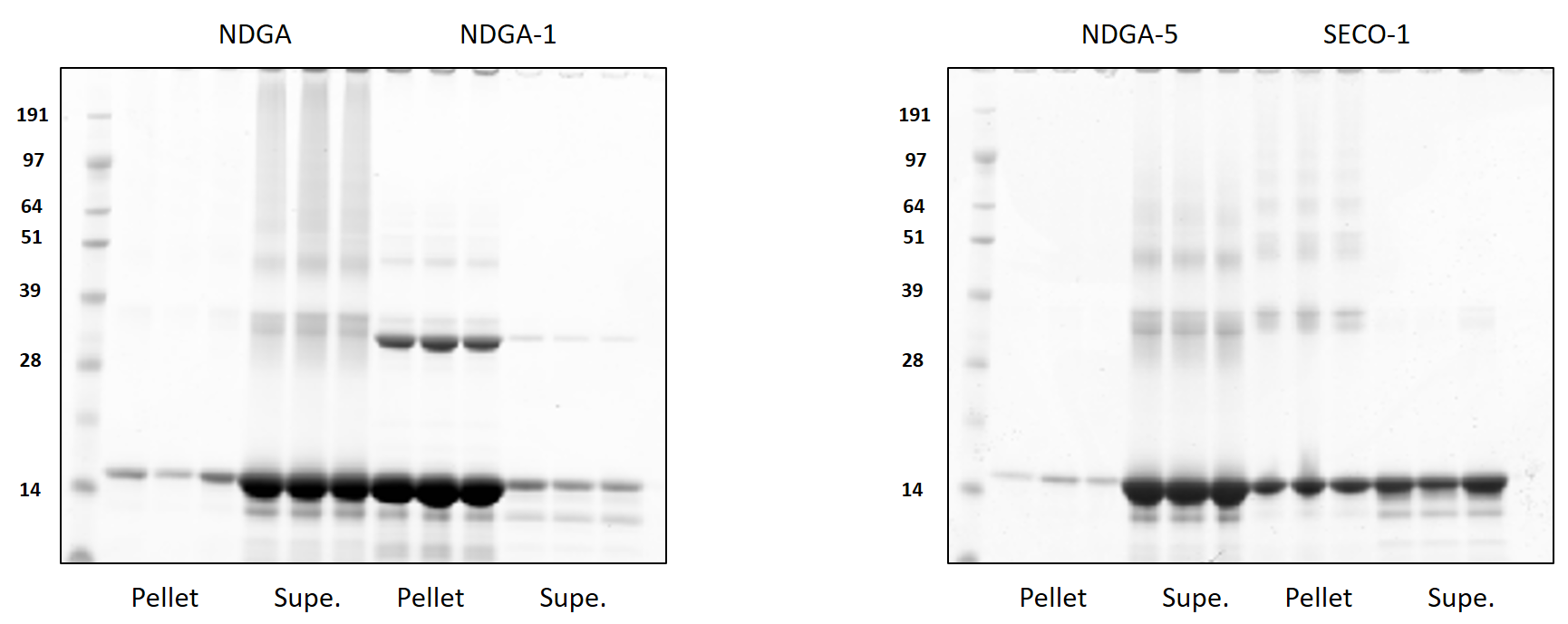


Colloidal

A


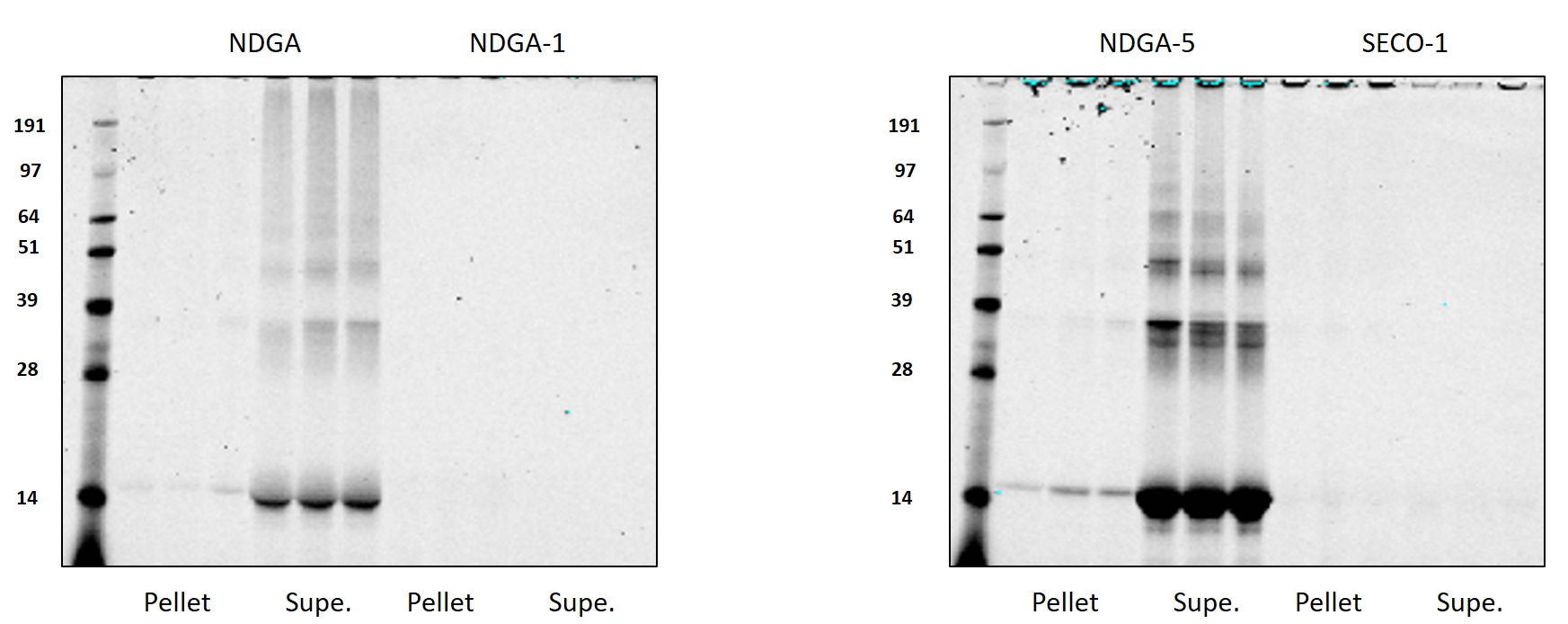


nIRF

B

Supplementary Figure S4. Colloidal and nIRF images of α-synuclein aggregation in the presence of NDGA analogs. **(a)** Colloidal images of gels after separation by centrifugation. **(b)** Near-infrared images of the same gels before colloidal staining, n=3.


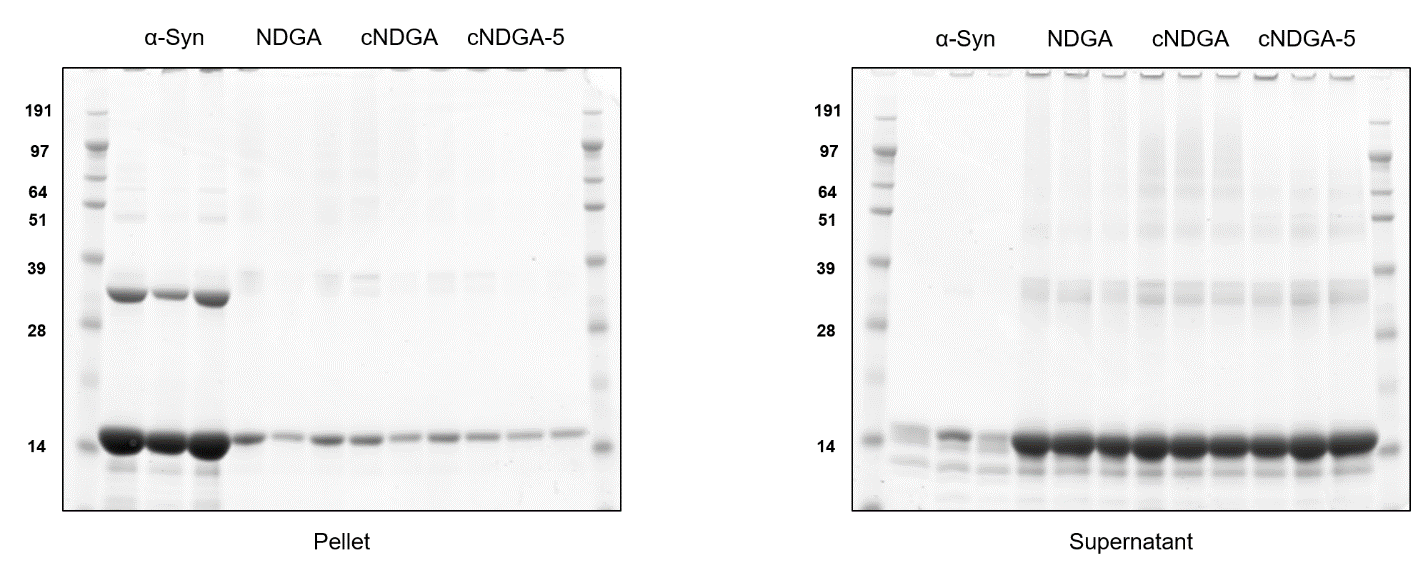


Colloidal

A

B


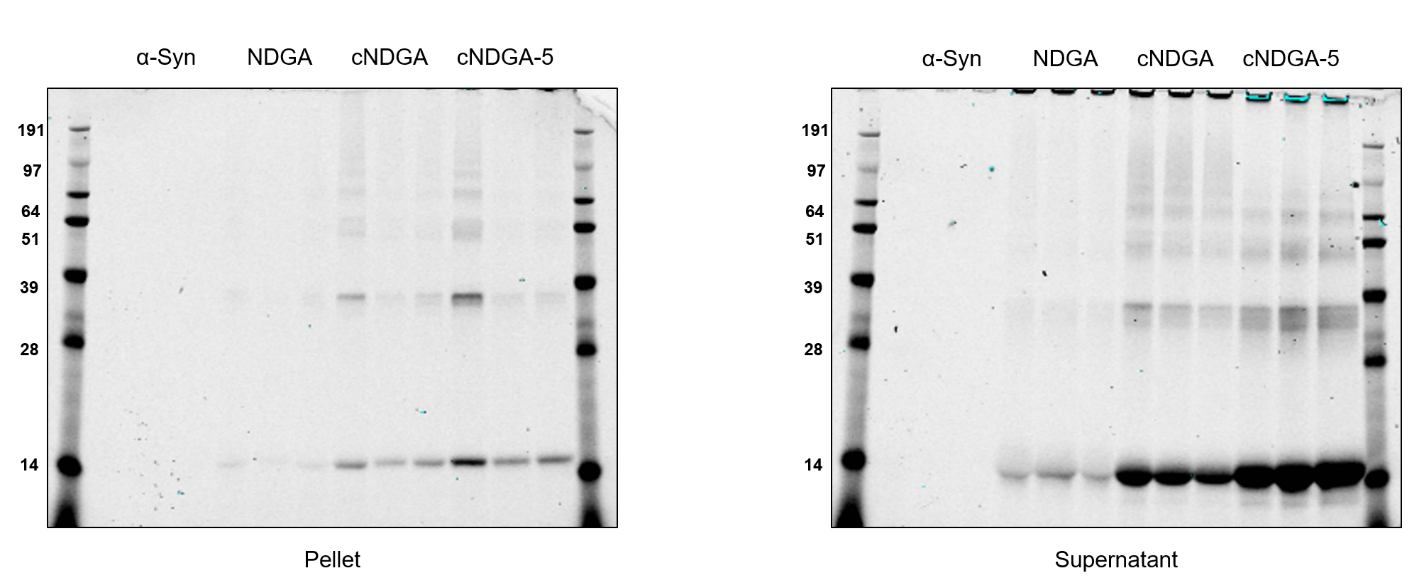


nIRF

Supplementary Figure S5. Colloidal and nIRF images of α-synuclein aggregation in the presence of cyclized NDGA analogs. **(a)** Colloidal images of gels after separation by centrifugation. **(b)** Near-infrared images of the same gels before colloidal staining, n=3.


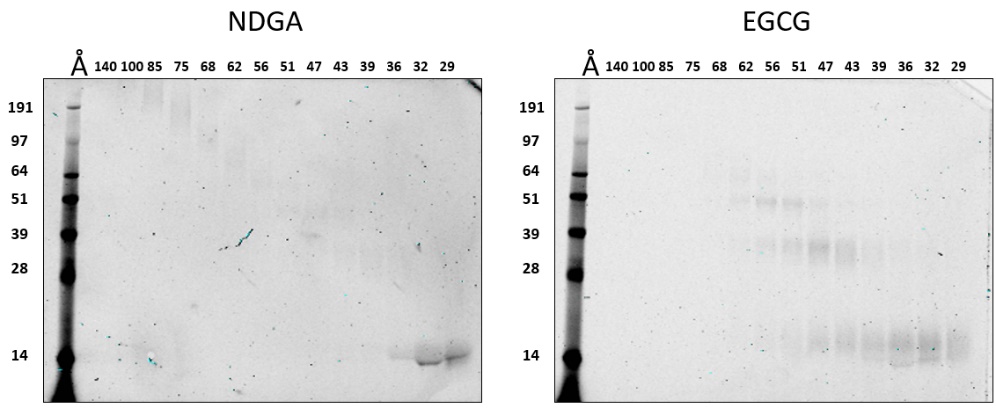


B

A


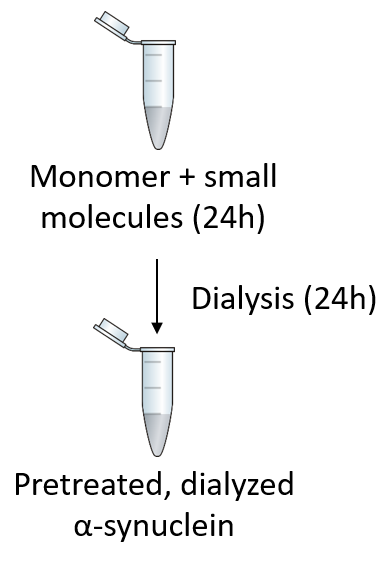


C


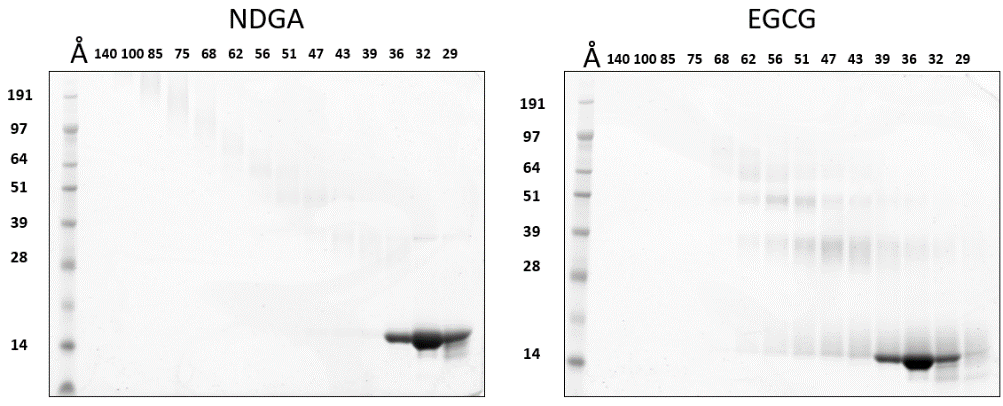


Supplementary Figure S6. NDGA pretreatment produces nIRF positive α-synuclein monomers. **(a)** Workflow for CD analysis and aggregation. **(b)** Near-infrared fluorescence images of NDGA and EGCG pretreated, dialyzed α-synuclein separated by native-state size-exclusion chromatography. **(c)** Colloidal staining of the same gels shown in panel b.

A





B

Supplementary Figure S7. Electrospray mass spectrometry characterization of α-synuclein-NDGA interaction. (a) Nano-ESI-MS spectra in positive-ion mode of 30 μM α-synuclein in the absence and presence of NDGA at 90 μM and 140 μM. (b) Magnification of the spectra in the Upper panel. The number of NDGA molecules bound to α-synuclein monomers is indicated by red numbers. Dimer-specific peaks of α-synuclein are labeled as “D”. Charge states are indicated by black numbers. Nano-ESI-MS spectra were collected as described in Konijnenberg et al.^49^


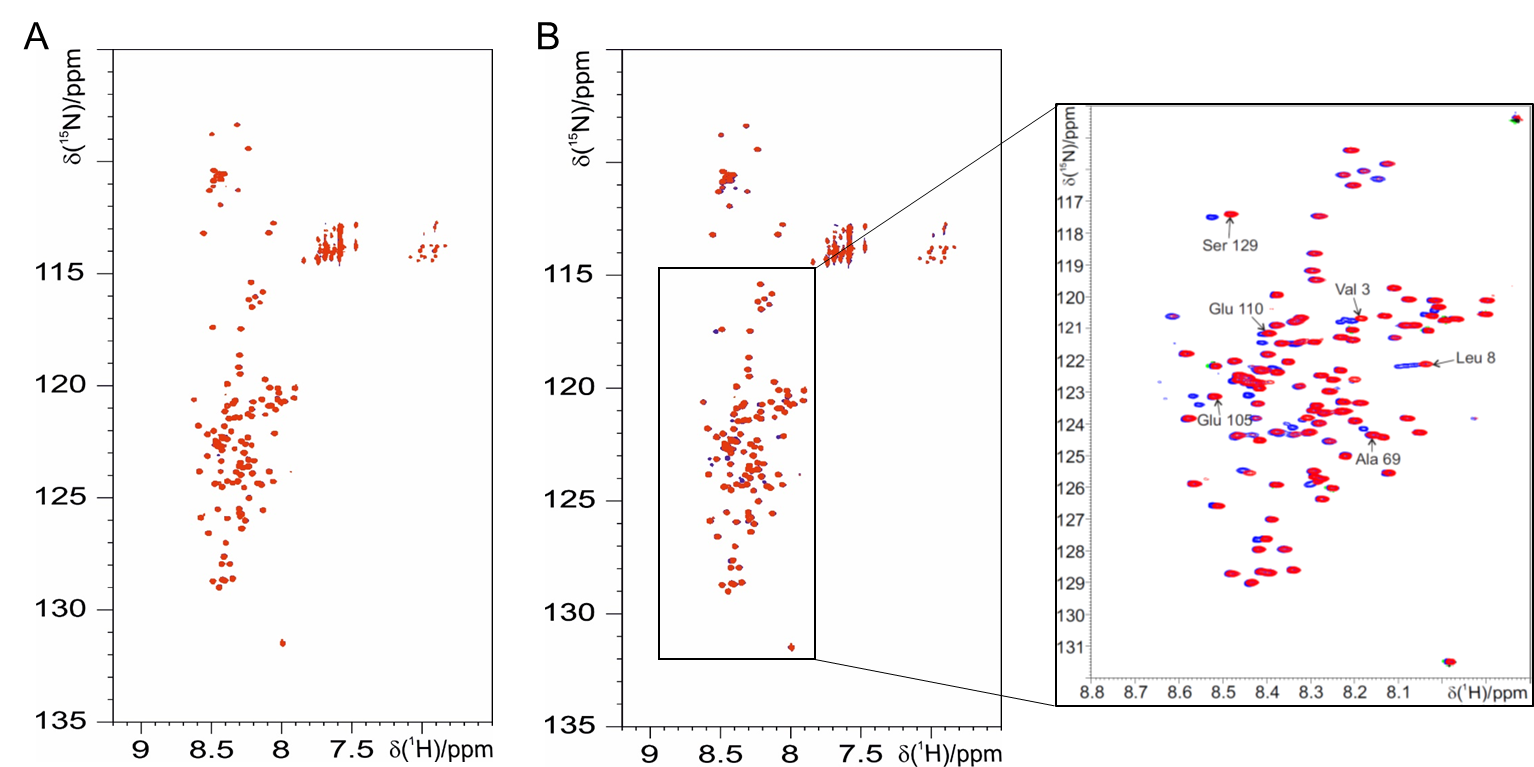


Supplementary Figure S8. 2D NMR spectra comparing α-synuclein treated with 1:1 NDGA for 24 hours (red), with **(a)** α-synuclein treated with 3:1 NDGA for 24 hours (blue), and **(b)** α-synuclein treated with 1:1 NDGA for 10 days (blue).


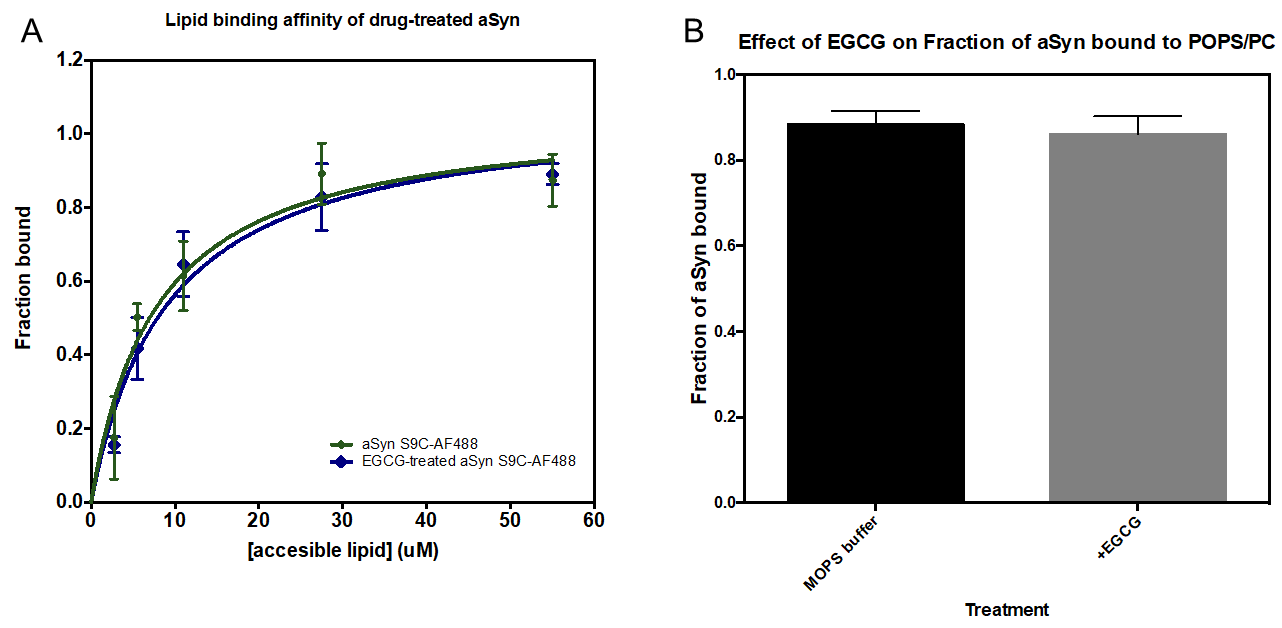


Supplementary Figure S9. EGCG does not disrupt lipid binding. (A) α-Synuclein was incubated 1:1 with EGCG or solvent alone for 24 hours before fluorescence correlation spectroscopy in the presence of POPS:POPC vesicles at the indicated concentrations. (n = 3). (B) Addition of NDGA did not displace fluorescently labeled α-synuclein from POPS:POPC vesicles. (n = 3).


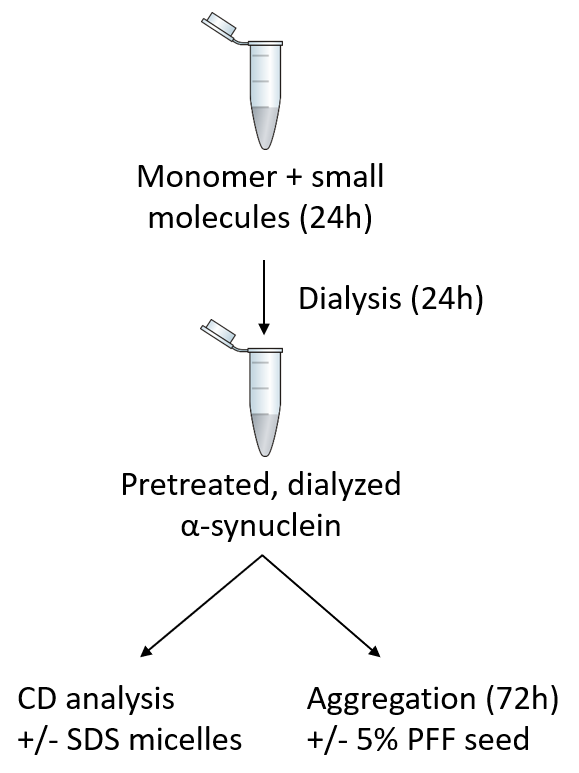


Supplementary Figure S10. Workflow for analysis of pretreated, dialyzed α-synuclein

Supplementary
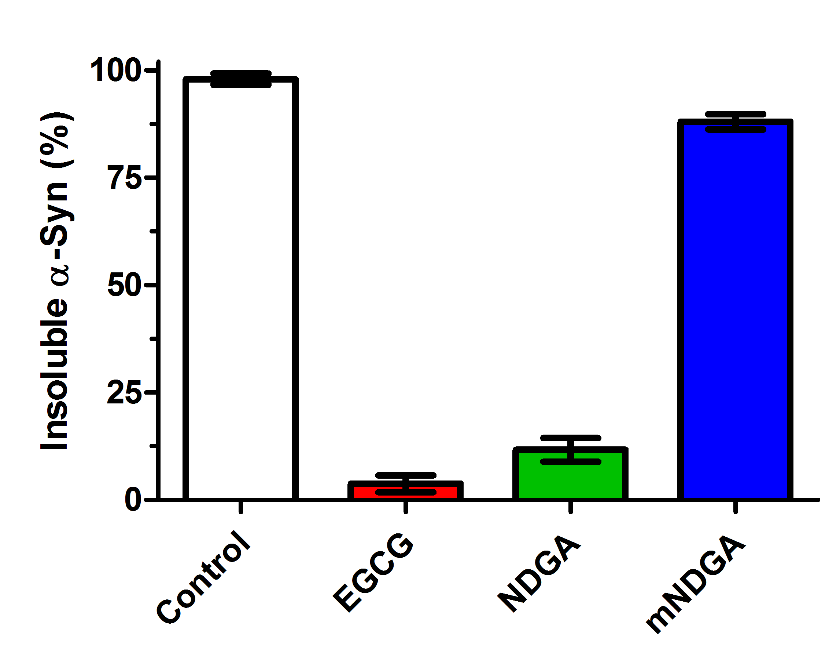
Figure S11. NDGA pretreatment inhibits α-synuclein aggregation despite 14 days under aggregation conditions. α-Synuclein was incubated 1:1 with small molecules for 24 hours then dialyzed against PBS for 24 hours. After aggregation for 14 days, PBS-insoluble α-synuclein was separated by centrifugation (21k g for 10 min). Soluble and insoluble fractions were boiled in SDS, run by SDS-PAGE, and colloidal stained. α-Synuclein in each fraction was quantified by in-gel densitometry. (n = 3)


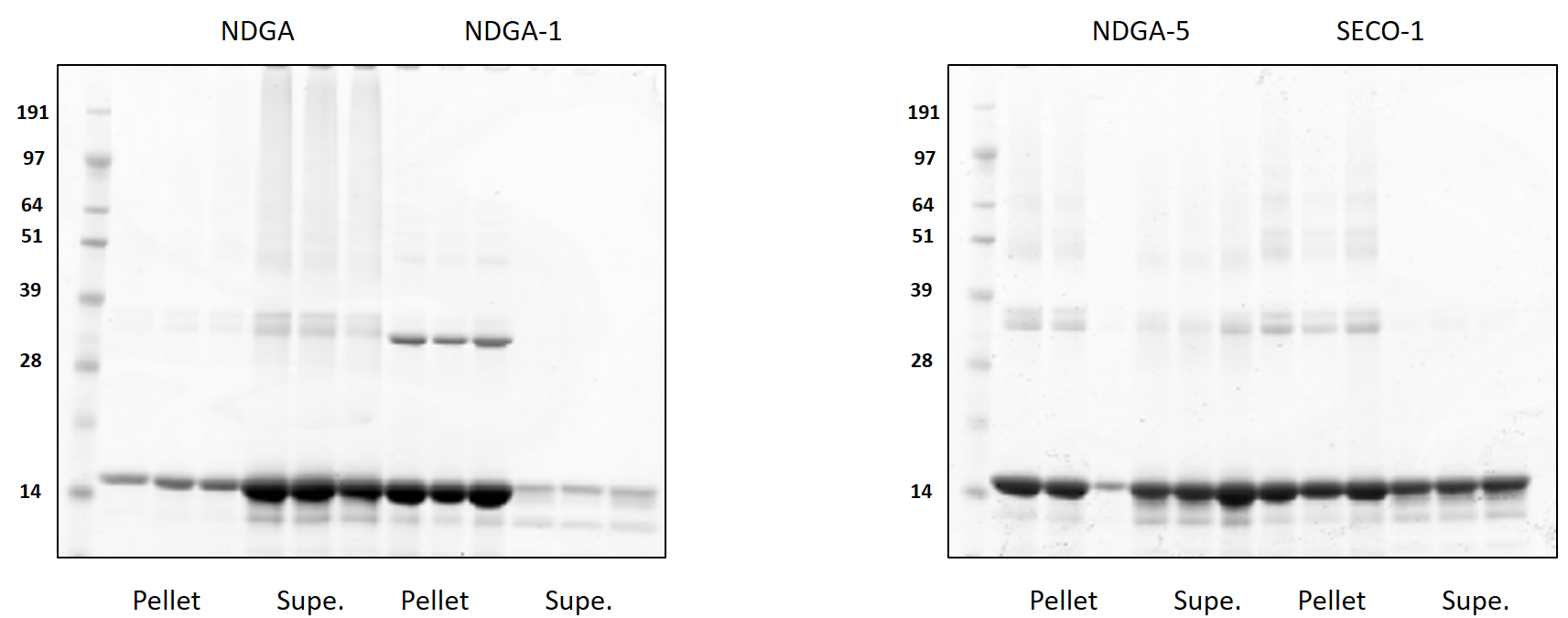


Colloidal

A


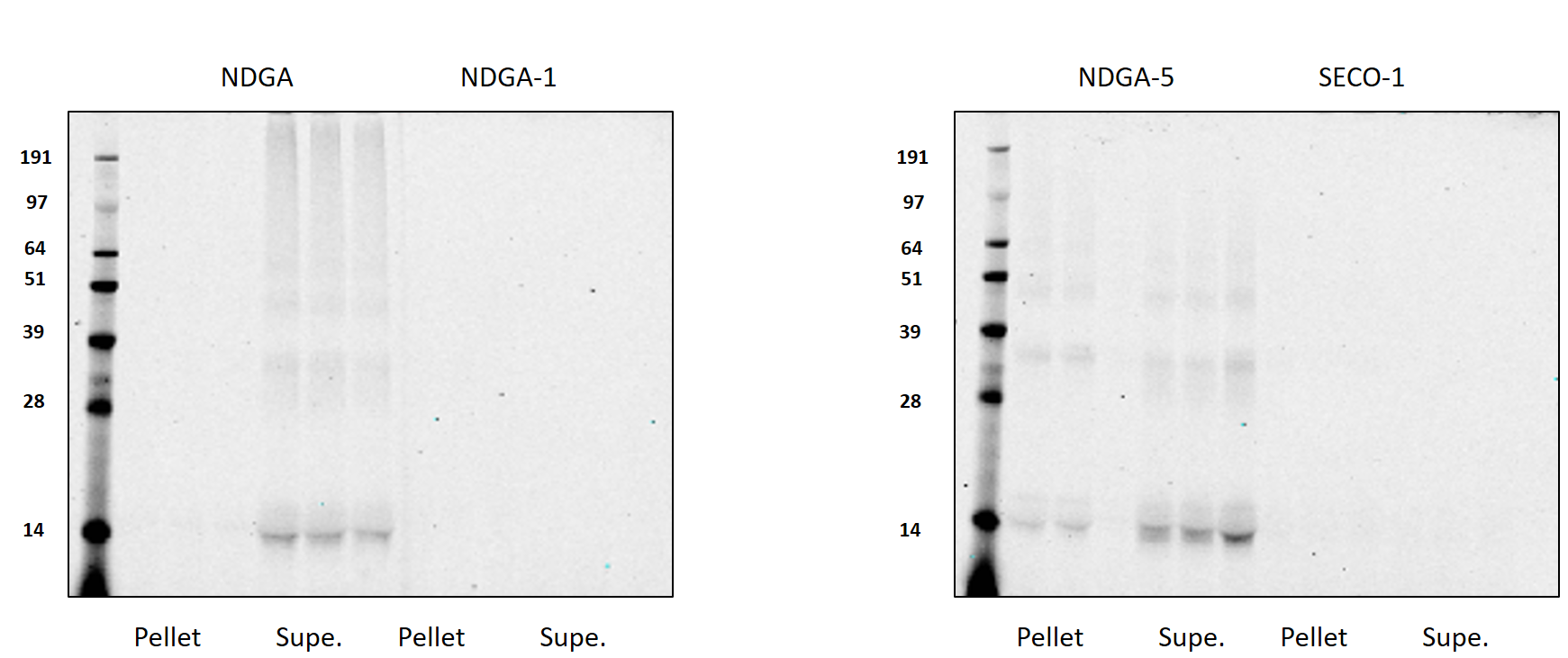


nIRF

B

Supplementary Figure S12. Colloidal and nIRF images of NDGA analog pretreated, dialyzed α-synuclein aggregation **(a)** Colloidal images of gels after separation by centrifugation. **(b)** Near-infrared images of the same gels before colloidal staining, n=3.


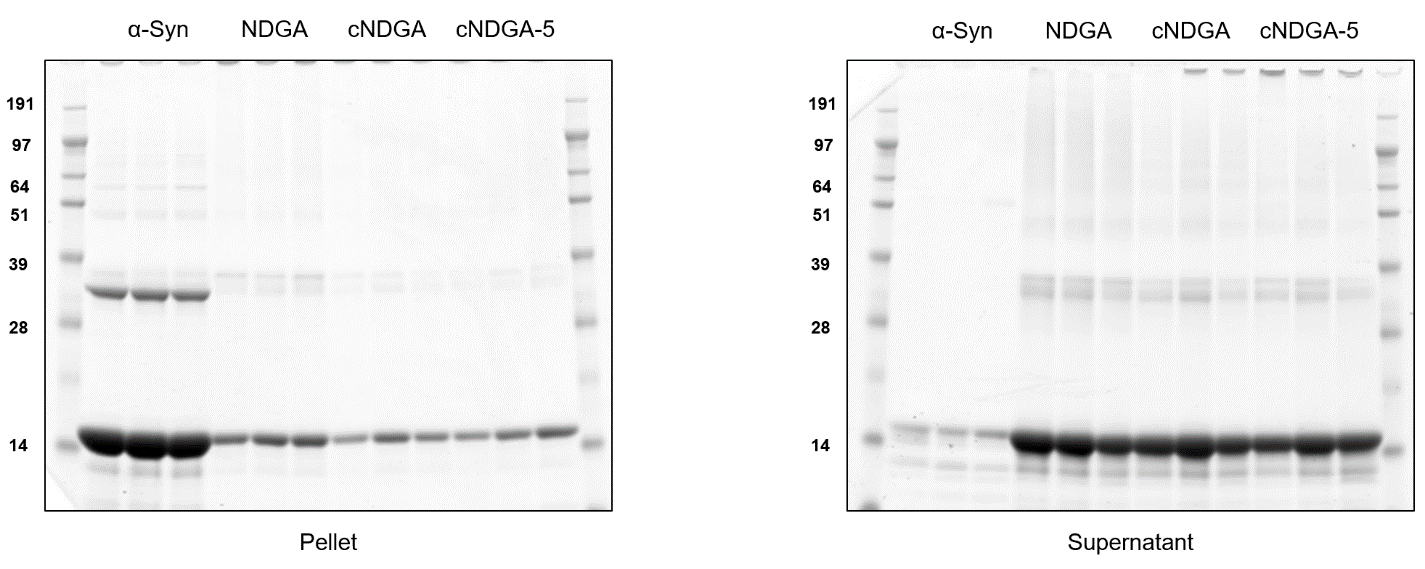


Colloidal

A

B


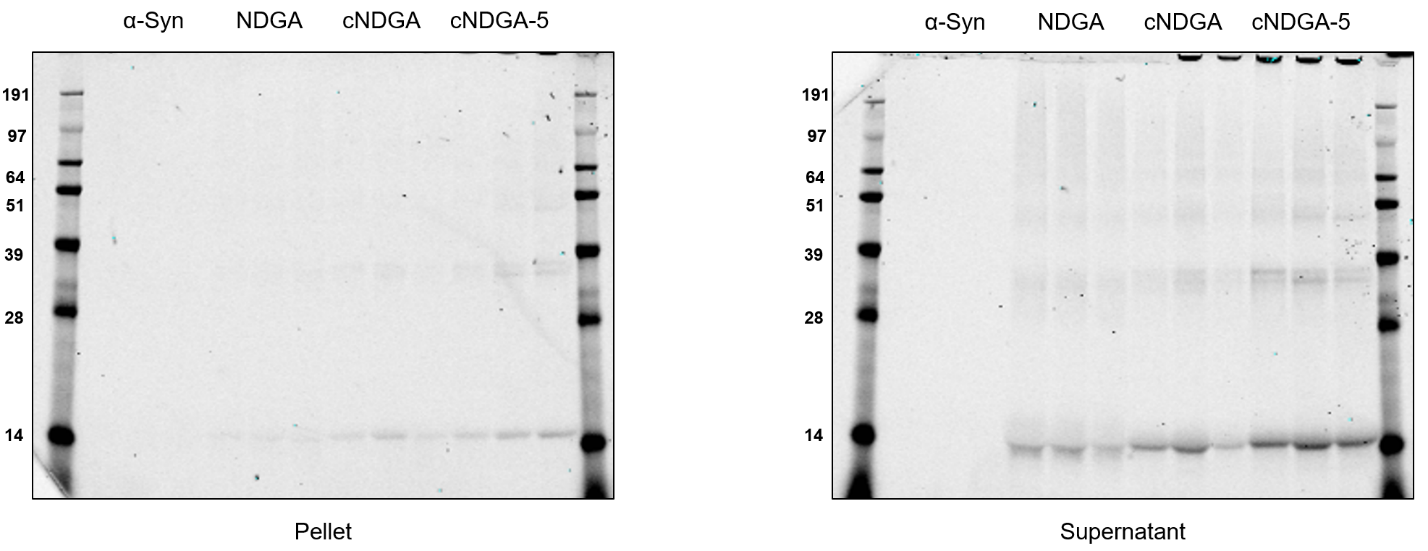


nIRF

Supplementary Figure S13. Colloidal and nIRF images of cyclized NDGA analog pretreated, dialyzed α-synuclein aggregation **(a)** Colloidal images of gels after separation by centrifugation. **(b)** Near-infrared images of the same gels before colloidal staining, n=3.


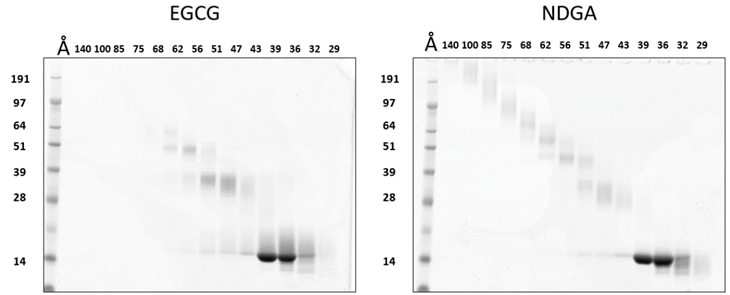


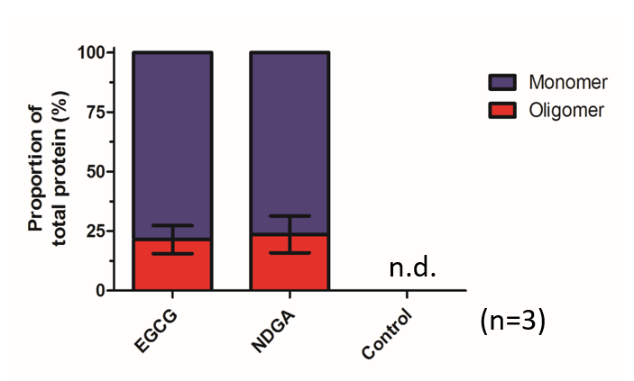


B


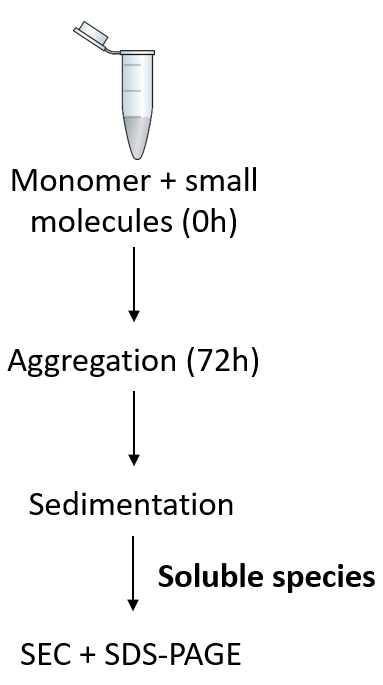


A

C


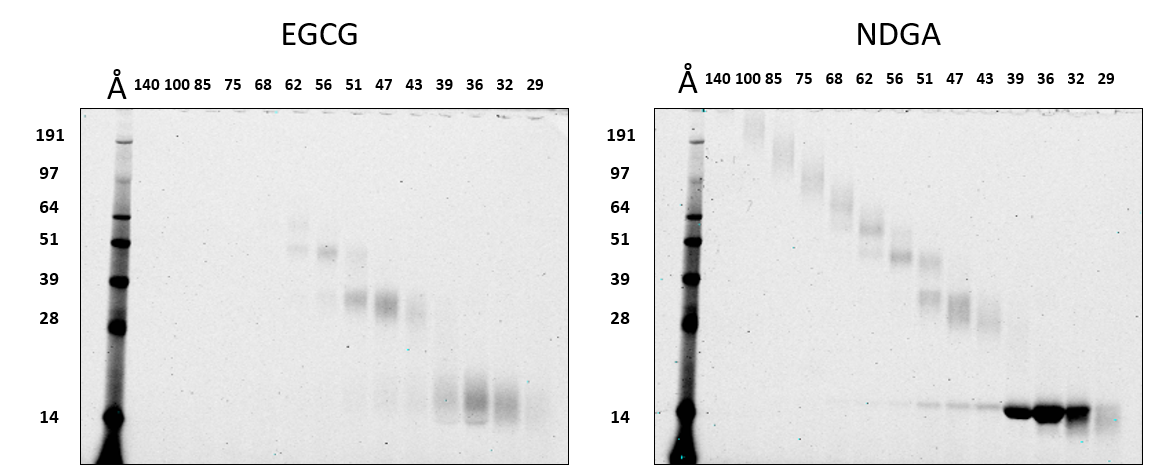


Supplementary Figure S14. nIRF positive monomers are the predominant product of α-synuclein aggregation in the presence of EGCG and NDGA. (a) Workflow (b) Colloidal (c) Densitometry (d) nIRF (n=3).

D


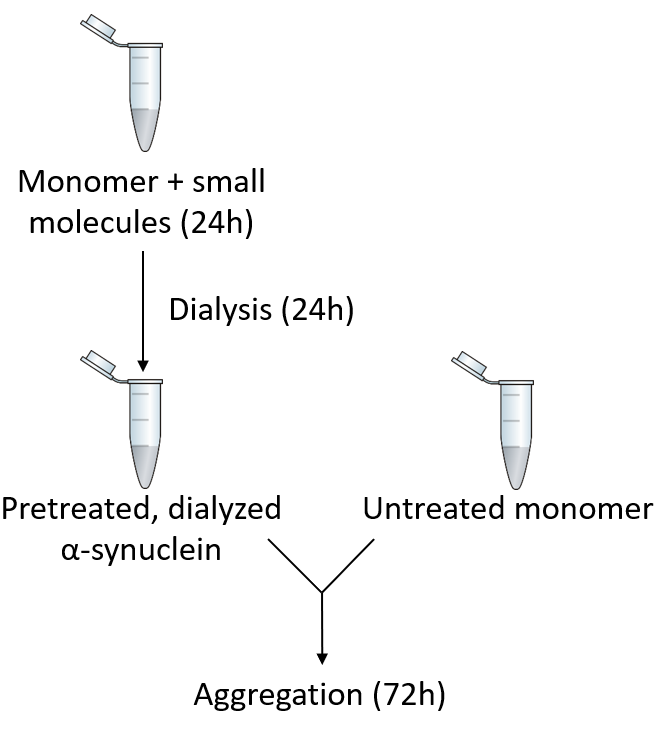


Supplementary Figure S15. Workflow for aggregation of combined mixtures of pretreated, dialyzed α-synuclein and untreated α-synuclein.

Supplementary Figure S16. EGCG reduces α-synuclein-driven neurodegeneration. *C. elegans* were treated with vehicle (dH_2_O) or 50µM EGCG in NGM agar on days 0-3, 5, and 7. Dopamine neurons were scored on day 8. Worms were scored as normal if no degenerative phenotypes (broken dendritic process, cell body loss, dendritic blebbing, or a missing neuron) were observed. Data represented as mean + SEM.; n=3 replicates with 30 worms per replicate; t-test; **p= 0.0023
